# Spatial multi-omics characterizes GPR35-relevant lipid metabolism signatures across liver zonation in MASLD

**DOI:** 10.1093/lifemeta/loae021

**Published:** 2024-05-31

**Authors:** Wuxiyar Otkur, Yiran Zhang, Yirong Li, Wenjun Bao, Tingze Feng, Bo Wu, Yaolu Ma, Jing Shi, Li Wang, Shaojun Pei, Wen Wang, Jixia Wang, Yaopeng Zhao, Yanfang Liu, Xiuling Li, Tian Xia, Fangjun Wang, Di Chen, Xinmiao Liang, Hai-long Piao

**Affiliations:** Key Laboratory of Phytochemistry and Natural Medicines, Dalian Institute of Chemical Physics, Chinese Academy of Sciences, Dalian, Liaoning 116023, China; School of Life Science and Biopharmaceutics, Shenyang Pharmaceutical University, Shengyang, Liaoning 110016, China; Key Laboratory of Phytochemistry and Natural Medicines, Dalian Institute of Chemical Physics, Chinese Academy of Sciences, Dalian, Liaoning 116023, China; University of Chinese Academy of Sciences, Beijing 100049, China; Key Laboratory of Phytochemistry and Natural Medicines, Dalian Institute of Chemical Physics, Chinese Academy of Sciences, Dalian, Liaoning 116023, China; University of Chinese Academy of Sciences, Beijing 100049, China; Key Laboratory of Phytochemistry and Natural Medicines, Dalian Institute of Chemical Physics, Chinese Academy of Sciences, Dalian, Liaoning 116023, China; University of Chinese Academy of Sciences, Beijing 100049, China; Key Laboratory of Phytochemistry and Natural Medicines, Dalian Institute of Chemical Physics, Chinese Academy of Sciences, Dalian, Liaoning 116023, China; University of Chinese Academy of Sciences, Beijing 100049, China; Key Laboratory of Phytochemistry and Natural Medicines, Dalian Institute of Chemical Physics, Chinese Academy of Sciences, Dalian, Liaoning 116023, China; Key Laboratory of Phytochemistry and Natural Medicines, Dalian Institute of Chemical Physics, Chinese Academy of Sciences, Dalian, Liaoning 116023, China; University of Chinese Academy of Sciences, Beijing 100049, China; Key Laboratory of Phytochemistry and Natural Medicines, Dalian Institute of Chemical Physics, Chinese Academy of Sciences, Dalian, Liaoning 116023, China; Department of Biochemistry & Molecular Biology, School of Life Sciences, China Medical University, Shenyang, Liaoning 110122, China; Key Laboratory of Phytochemistry and Natural Medicines, Dalian Institute of Chemical Physics, Chinese Academy of Sciences, Dalian, Liaoning 116023, China; Laboratory of High-Resolution Mass Spectrometry Technologies, Dalian Institute of Chemical Physics, Chinese Academy of Sciences, Dalian, Liaoning 116023, China; Key Laboratory of Phytochemistry and Natural Medicines, Dalian Institute of Chemical Physics, Chinese Academy of Sciences, Dalian, Liaoning 116023, China; University of Chinese Academy of Sciences, Beijing 100049, China; Key Laboratory of Phytochemistry and Natural Medicines, Dalian Institute of Chemical Physics, Chinese Academy of Sciences, Dalian, Liaoning 116023, China; Key Laboratory of Phytochemistry and Natural Medicines, Dalian Institute of Chemical Physics, Chinese Academy of Sciences, Dalian, Liaoning 116023, China; Key Laboratory of Phytochemistry and Natural Medicines, Dalian Institute of Chemical Physics, Chinese Academy of Sciences, Dalian, Liaoning 116023, China; Key Laboratory of Phytochemistry and Natural Medicines, Dalian Institute of Chemical Physics, Chinese Academy of Sciences, Dalian, Liaoning 116023, China; Key Laboratory of Phytochemistry and Natural Medicines, Dalian Institute of Chemical Physics, Chinese Academy of Sciences, Dalian, Liaoning 116023, China; Key Laboratory of Phytochemistry and Natural Medicines, Dalian Institute of Chemical Physics, Chinese Academy of Sciences, Dalian, Liaoning 116023, China; Key Laboratory of Phytochemistry and Natural Medicines, Dalian Institute of Chemical Physics, Chinese Academy of Sciences, Dalian, Liaoning 116023, China; University of Chinese Academy of Sciences, Beijing 100049, China; Key Laboratory of Phytochemistry and Natural Medicines, Dalian Institute of Chemical Physics, Chinese Academy of Sciences, Dalian, Liaoning 116023, China; University of Chinese Academy of Sciences, Beijing 100049, China; Key Laboratory of Phytochemistry and Natural Medicines, Dalian Institute of Chemical Physics, Chinese Academy of Sciences, Dalian, Liaoning 116023, China; University of Chinese Academy of Sciences, Beijing 100049, China; Key Laboratory of Phytochemistry and Natural Medicines, Dalian Institute of Chemical Physics, Chinese Academy of Sciences, Dalian, Liaoning 116023, China; University of Chinese Academy of Sciences, Beijing 100049, China; Department of Biochemistry & Molecular Biology, School of Life Sciences, China Medical University, Shenyang, Liaoning 110122, China

**Keywords:** GPR35, MASLD, liver zonation, spatial transcriptomics, spatial metabolomics

## Abstract

Metabolic dysfunction-associated steatotic liver disease (MASLD) is a metabolic disease that can progress to metabolic dysfunction-associated steatohepatitis (MASH), cirrhosis, and cancer. The zonal distribution of biomolecules in the liver is implicated in mediating the disease progression. Recently, G-protein-coupled receptor 35 (GPR35) has been highlighted to play a role in MASLD, but the precise mechanism is not fully understood, particularly, in a liver-zonal manner. Here, we aimed to identify spatially distributed specific genes and metabolites in different liver zonation that are regulated by GPR35 in MASLD, by combining lipid metabolomics, spatial transcriptomics (ST), and spatial metabolomics (SM). We found that GPR35 influenced lipid accumulation, inflammatory and metabolism-related factors in specific regions, notably affecting the anti-inflammation factor ELF4 (E74 like E-twenty six (ETS) transcription factor 4), lipid homeostasis key factor CIDEA (cell death-inducing DNA fragmentation factor alpha (DFFA)-like effector A), and the injury response-related genes *SAA1/2/3* (serum amyloid A1/2/3), thereby impacting MASLD progression. Furthermore, SM elucidated specific metabolite distributions across different liver regions, such as C10H11N4O7P (3ʹ,5ʹ-cyclic inosine monophosphate (3ʹ,5ʹ-IMP)) for the central vein, and this metabolite significantly decreased in the liver zones of *GPR35*-deficient mice during MASLD progression. Taken together, GPR35 regulates hepatocyte damage repair, controls inflammation, and prevents MASLD progression by influencing phospholipid homeostasis and gene expression in a zonal manner.

## Introduction

Fatty liver disease, also known as hepatic steatosis, is a growing global health concern with significant implications for morbidity and mortality. It is characterized by abnormal accumulation of lipids within hepatocytes, leading to liver dysfunction and potential progression to more severe conditions, such as metabolic dysfunction-associated steatohepatitis (MASH) and liver fibrosis. Despite its increasing prevalence and impact on public health, the underlying molecular mechanisms driving the development and progression of fatty liver disease remain incomplete.

The liver zonation, which involves the division of the liver into distinct metabolic zones, much like an assembly line that assigns specific metabolic tasks to hepatocytes in different lobular zones, plays a crucial role in coordinating complex, complementary, or opposing multistep processes in the liver [1]. Zonal heterogeneity is well-documented in the context of hepatic metabolism, with various zones exhibiting variations in gene expression profiles and metabolic activities [2]. Understanding the molecular basis of liver zonation is essential for unraveling the intricate mechanisms involved in the pathogenesis of fatty liver disease.

G-protein-coupled receptor 35 (GPR35) is involved in various physiological processes, notably inflammation and metabolism. Recent studies have shed light on the critical physiological and pathological roles of GPR35. For instance, it has been shown to promote neutrophil transmigration to infected sites [3] and facilitate the remodeling of mitochondrial function, thereby preventing ischemic condition [4]. Moreover, GPR35 can prevent cell growth arrest and apoptosis under challenging culture conditions [5] and promote vascular smooth muscle cell migration and proliferation via the Rho kinase signaling axis [6]. Additionally, activation of its agonist has demonstrated potential in protecting against dextran sodium sulfate (DSS)-induced colitis [7], highlighting *GPR35* as a pro-survival gene that regulates immunity and cell survival. Furthermore, GPR35 plays a role in modulating lipid metabolism, thermogenesis, and inflammation in adipose tissue to maintain energy homeostasis [3, 8]. While previous studies have implicated GPR35 in the context of metabolic dysfunction-associated steatotic liver disease (MASLD) and MASH [9–12], its precise function in fatty liver disease and how it modulates metabolism in specific liver zones remain to be fully characterized.

In recent years, cutting-edge technologies, such as single-cell RNA (scRNA) sequencing, spatial transcriptomics (ST), and spatial metabolomics (SM), have revolutionized our capacity to study the spatial organization of tissues at the molecular level [13–16]. Here, we have integrated SM and ST approaches to investigate liver zonation and elucidate the mechanistic role of GPR35 in the regulation of fatty liver disease. By combining spatial multi-omics, we attempted to uncover the spatial distribution of metabolites and gene expressions within different liver zones under high-fat diet (HFD) conditions. This study has revealed zonal heterogeneity and potential correlations between metabolite composition and gene expression profiles in an MASLD mouse model. Our primary focus is on illuminating the role of GPR35 in influencing liver zonation and its implications for the development and progression of fatty liver disease. Ultimately, this research holds the promise of offering valuable insights into the underlying mechanisms governing liver zonation and the molecular basis of GPR35 involving in regulating lipid metabolism and fatty liver disease.

## Results

### Accumulating lipid metabolism in *Gpr35* knock-out (KO) mouse liver

To investigate GPR35-regulated biomolecules in liver zonation within the MASLD model, we segregated wild-type (WT) and *Gpr35* KO mice into normal-chow-diet (ND) and HFD conditions (Supplementary Fig. S1a and b). Subsequently, *Gpr35* KO mice exhibited a significant increase in body weight compared to WT mice after 10 weeks under HFD conditions, while both WT and *Gpr35* KO mice remained similar under ND conditions (Supplementary Fig. S1b). Additionally, intraperitoneal glucose tolerance test (IPGTT) revealed higher glucose levels in *Gpr35* KO mice compared to WT mice following the intervention (Supplementary Fig. S1c). After 14 weeks, we sacrificed the mice and observed that *Gpr35* KO mice had a more substantial fat content in inguinal white adipose tissue (iWAT), epididymal white adipose tissue (eWAT), and retroperitoneal white adipose tissue (rWAT) (Supplementary Fig. S1d–h). Furthermore, *Gpr35* KO mice exhibited increased hepatic steatosis compared to WT mice (Supplementary Fig. S1i). These results suggest that *Gpr35* deficiency exacerbates the progression of MASLD.

**Figure 1:**
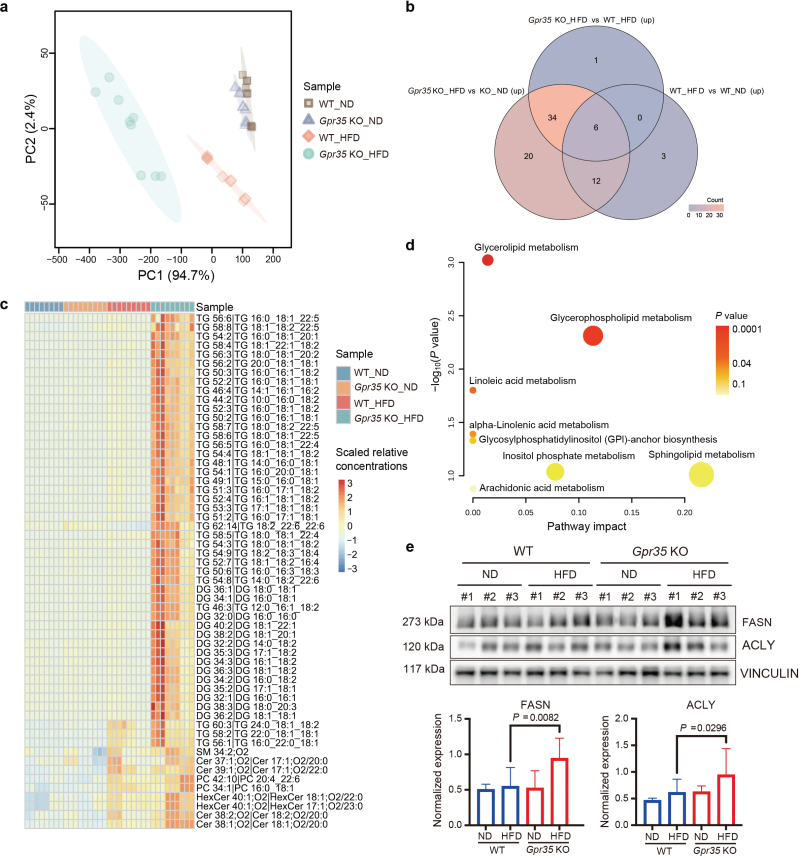
Lipidomics analysis of WT and *Gpr35* KO mouse livers under ND and HFD conditions. (a) PCA plot of lipid concentrations of samples from four different conditions were visualized by the MetaboAnalyst website. (b) Venn diagram of differential metabolite analysis for *Gpr35* KO_HFD versus WT_HFD, *Gpr35* KO_HFD versus *Gpr35* KO_ND, and WT_HFD versus WT_ND. (c) Heatmap of the top differential metabolites in *Gpr35* KO_HFD group compared with samples of other experimental conditions. (d) Metabolism pathways enriched by significantly changed metabolites between *Gpr35* KO_HFD and WT_HFD samples. The analysis was performed on the MetaboAnalyst website. The X-axis shows the pathway impact, and the Y-axis shows the *P* value after the log_10_ transformation. (e) Immunoblotting analysis of indicated antibodies in WT or *Gpr35* KO mouse liver tissues under ND or HFD conditions (upper panel), and normalized expression of FASN and ACLY (bottom panel).

To describe the disordered metabolism in the MASLD mouse model, high-throughput lipid-omics was performed to detect the differentially altered metabolites in WT and *Gpr35* KO mouse livers under ND and HFD conditions. In a principal component analysis (PCA) model, the samples from different conditions were located together (Fig. 1a). Despite similar metabolic characteristics of WT and *Gpr35* KO groups obtained from the PCA model model, a discriminatory trend was presented between ND and HFD groups (Fig. 1a). Compared with the other three groups, 20 metabolites were significantly altered in the *Gpr35* KO_HFD livers (Fig. 1b). The upregulated metabolites were mainly triglycerides (TGs) and diglycerides (DGs) (Fig. 1c). A further pathway enrichment analysis showed that glycerolipid metabolism, glycerophospholipid metabolism, lipid storage, and lipid droplet signaling were enriched in *Gpr35* KO_HFD livers (Fig. 1d; Supplementary Fig. S1j). In addition, we analyzed the lipid metabolism-related protein expressions and found that ATP citrate lyase (ACLY) and fatty acid synthase (FASN) were highly expressed in *Gpr35* KO_HFD livers compared with the other three groups (Fig. 1e). These results confirmed that the contents of lipid metabolism were significantly upregulated in *Gpr35* KO_HFD livers, but what caused the abnormal upregulation of lipidome in *Gpr35* KO_HFD livers remains unclear.

**Figure 2:**
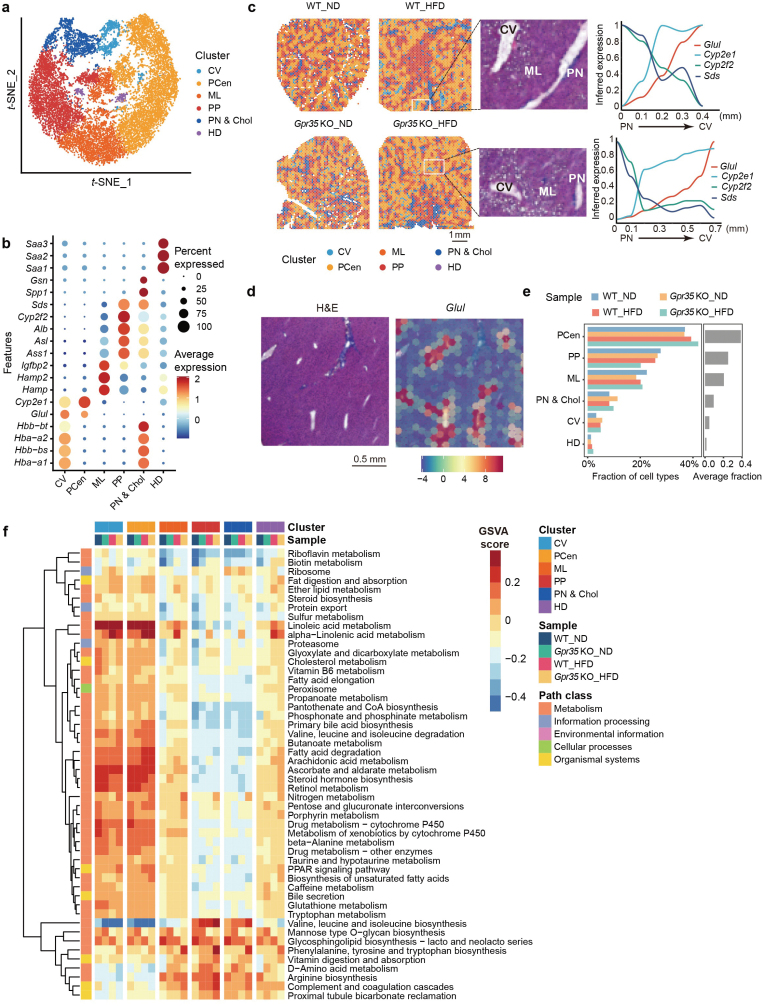
Spatial mRNA features in mouse liver zonation under ND and HFD conditions. (a) *t*-SNE plot colored by the distinct liver zonation clusters. (b) Dot plot of the expressions of marker genes for different liver zonation clusters. The colors and point sizes represent the average expression and percentage of specific marker genes in each cluster. (c) Spatial distributions of distinct liver zonation clusters (left panel) and corresponding high-magnification images of distinct histological regions, including the PN and CV, alongside spatial trajectories illustrating the expression patterns of marker genes (right panel). (d) Representative magnified image from WT_ND in c, and different colors indicate GLUL expression in distinct liver zonation. (e) The proportions of different liver zonation clusters within each sample. The Y-axis represents the liver zonation, and the X-axis represents the percentage. The colors represent sample types. (f) KEGG-based mean GSVA enrichment score of spatial spots within each cluster in four different samples. For each sample, normalization and standardization were performed separately, followed by GSVA analysis. Subsequently, the standard deviation changes were calculated after merging across different samples and clusters. Only the pathways displaying the top 50 standard deviation changes in values across different samples and clusters are displayed.

Next, to explore the impact of GPR35 on gene expression and the distribution of metabolites within liver zonation at the spatial single-cell level, we conducted an integrative analysis of multiple spatial omics data, encompassing ST and SM. In each experimental group, we collected two adjacent samples for concurrent ST and SM analysis. This approach was aimed at identifying spatial gene zonation features and employing spatial mass spectrum imaging to investigate the spatial distribution of metabolites and lipids. The spatially resolved transcriptome analysis was carried out using the 10× Genomics platform, yielding an average of 4095 data points for each sample (Fig. 2a; Supplementary Fig. S2a). By combining these two spatial omics methodologies, we attempted to obtain a comprehensive understanding of the biomolecular changes associated with GPR35 and its role in responding to MASLD.

### ST unveils molecular characteristics of liver zonation in mice

The liver is an organ characterized by a well-defined histological structure, consisting of liver lobules. Traditionally, these lobules are described as having a central vein (CV) surrounded by six portal nodes (PNs), along with adjacent bile ducts (BDs). Next, to understand spatial histological structure, a *t*-distributed stochastic neighbor embedding (*t*-SNE) plot were performed and six distinct clusters were identified (Fig. 2a; Supplementary Fig. S2a), which exhibited zonation-like distribution (Fig. 2b and c; Supplementary Fig. S2b and c). The dot plot illustrated the expression levels of liver zonation marker genes in different clusters (Fig. 2b). This analysis confirmed the pericentral area (PCen or Zone 3) by the enriched expression of marker genes, glutamate-ammonia ligase (*Glul*) and cytochrome P450 2E1 (*Cyp2e1*), while the periportal area (PP or Zone 1) by the expression of specific marker genes, such as cytochrome P450 2F2 (*Cyp2f2*), serine dehydratase (*Sds*), argininosuccinate synthetase 1 (*Ass1*), argininosuccinate lyase (*Asl*), and albumin (*Alb*) [2, 17], and the middle lobule layer area (ML or Zone 2) by display of the marker genes hepcidin antimicrobial peptide (*Hamp*) and *Hamp2* expression (Fig. 2b). Additively, we identified spatially distinct and refined clusters, with the PCen and PP clusters further dividing into two sub-clusters (PCen1, PCen2 and PP1, PP2) (Supplementary Fig. S2b). Based on the observation of significant upregulation of hemoglobin genes *Hba-a1*, *Hba-a2*, *Hbb-bs*, and *Hbb-bt* [18], we confirmed the CV and PN & Chol (cholangiocyte) region (Fig. 2b). To further validate the zonal distribution of these clusters, we superimposed the ST data onto hematoxylin and eosin (H&E) stain images (Fig. 2c). To provide a clear representation of the histological characteristics of the portal vein and the CV within different samples, we selected regions with distinct histological features for magnification. The expression trends of region-specific feature genes were presented (Fig. 2c right panel). In parallel, we magnified the spatial expression of *Glul* across the liver zones in WT (Fig. 2d).

Intriguingly, a distinctive cluster was identified, which exhibited a significant upregulation of serum amyloid A1, A2, and A3 (*Saa1*, *Saa2*, and *Saa3*) (Fig. 2b). Since these genes have been associated with a poorer prognosis and are typically located in the invasive zone [19, 20] and associated with liver damage, we annotated this cluster as the hepatocyte damaged (HD). In contrast to the HD cluster, the other clusters demonstrated a spatial liver zonation connection, progressing from the CV, PCen, ML, and PP to PN [21], which aligned with the magnified region of WT_ND (Supplementary Fig. S2c). Additionally, considering the H&E annotations, the severity of lipid accumulation followed the pattern from WT_ND, *Gpr35 *KO_ND, and WT_HFD to *Gpr35 *KO_HFD (Supplementary Fig. S1i). Notably, the fraction of the HD and PCen clusters, which are associated with liver damage, increased proportionally with the degree of lipid accumulation (Fig. 2e) [22]. In comparison, the spatial distribution of PCen marker genes, including *Glul* and *Cyp2e1*, and PP marker genes, like *Sds* and *Cyp2f2*, across four different conditions are identical (Supplementary Fig. S2d).

For insight exploration of pathophysiological association between the zonally distributed clusters and the metabolic signaling pathways, we conducted gene set enrichment analysis using the Kyoto Encyclopedia of Genes and Genomes (KEGG) pathway datasets and observed a prevalence of metabolism-related pathways. Specifically, the CV and PCen clusters exhibited upregulation in pathways linked to fatty acid elongation, amino acid, glycolysis/gluconeogenesis, and lipoic acid metabolism (Supplementary Fig. S2e). Interestingly, only the transforming growth factor-β (TGF-β) signaling pathway was enriched in the ML cluster (Supplementary Fig. S2e). The HD cluster displayed significant expression in pathways such as cytokine, tumor necrosis factor (TNF), and TGF-β signaling pathways (Supplementary Fig. S2e). Additionally, while most signaling pathways demonstrated differential enrichment across liver zonation, the signaling pathways among them like fatty acid elongation, peroxisome proliferator-activated receptor (PPAR) signaling pathway, cholesterol metabolism, and bile secretion pathway exhibited increased enrichment in CV and PCen clusters compared to PP and PN & Chol (Fig. 2f; Supplementary Fig. S2f). In addition, to ensure that our findings are independent of previous studies, we performed pathway correlation analysis by integrating our transcriptomic data with the proteomic data sets [9]. Indeed, we did not find direct evidence that cholesterol homeostasis was regulated by GPR35 in both data sets (Supplementary Fig. S2g). However, retinal metabolism and steroid hormone biosynthesis were enriched, in which steroid hormone biosynthesis is related to cholesterol homeostasis (Supplementary Fig. S2g). In contrast, we discovered many other differentiated gene expressions, which were correlated with the regulation of immune response, inflammation, actin cytoskeleton remodeling, and other metabolic process, including fatty acid elongation, cholesterol metabolism, and bile secretion in our transcriptomic data sets (Supplementary Fig. S2g). These results suggested that different dietary conditions turn on different signaling pathways.

### Investigation of the spatial molecular expression in mouse liver histopathological patterns

**Figure 3:**
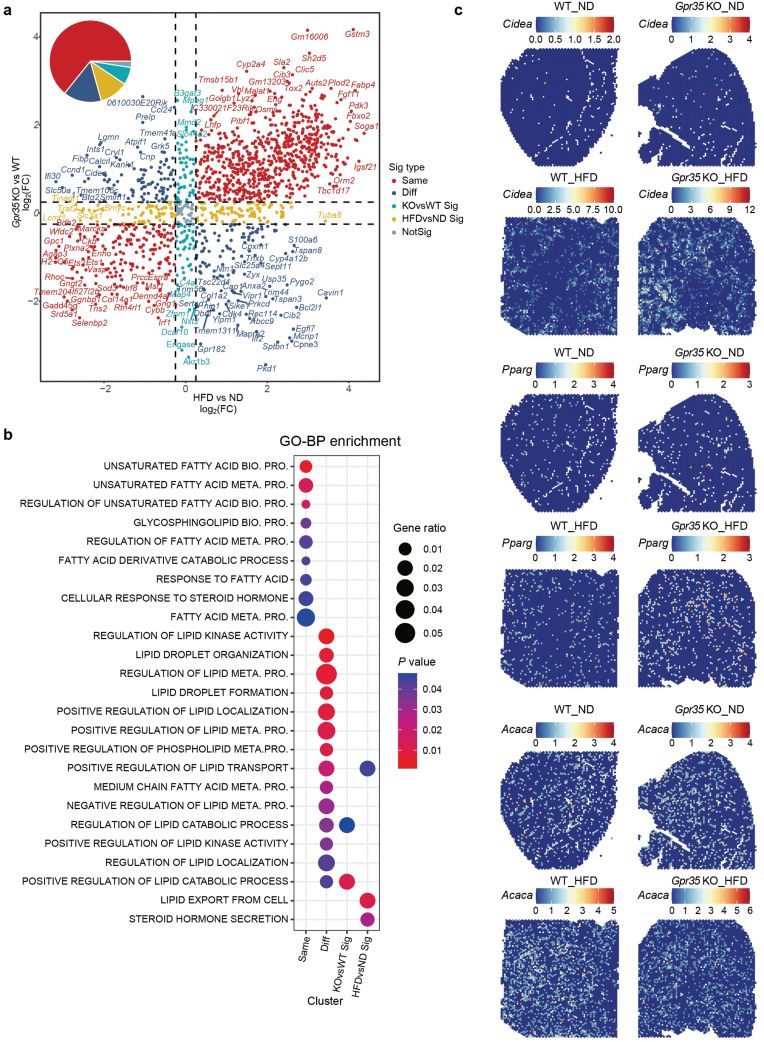
Gene expression commonness and differences between WT and *Gpr35* KO mouse livers under HFD conditions. (a) Scatter plot of the DEGs in two different comparison manners. The X-axis represents the average log_2_-transformed fold change (avg_log_2_(FC)) computed by comparing the HFD and ND liver tissues (HFDvsND). The Y-axis represents the avg_log_2_(FC) computed by comparing the *Gpr35* KO and WT liver tissues (KOvsWT). The point colors represent the significance type. (b) Gene ontology-biological processes (GO-BP) over-representation analysis based on four different types of DEGs. Adjusted *P* values (Benjamini and Hochberg correction method) are indicated by colors and gene ratios are represented by point sizes. Only significant pathways (*P* < 0.05) belonging to the “metabolism” or “signaling transduction” categories in the KEGG classification are displayed. The over-representation significance was examined based on hypergeometric distribution. PRO.: process; META.: metabolism. (c) Representative spatial distributions of genes *Cidea* (upper panel), *Pparg* (middle panel), and *Acaca* (lower panel) in WT and *Gpr35* KO mouse livers under ND or HFD conditions.

Examining the changes in gene expression within liver zonation under HFD conditions can offer valuable insights into the potential key molecular mechanisms driving the development and progression of MASLD. To investigate GPR35-regulated key factors in MASLD, we initially compared the gene expression profiles of WT and *Gpr35* KO mice (KOvsWT). We also compared the gene expression profiles between ND and HFD conditions (HFDvsND). Each gene underwent two sets of differential expression analyses, and the resulting average log_2_(fold change) (avg_log_2_(FC)) values were represented on the × and y axes, respectively (Fig. 3a; Supplementary Fig. S3a). Genes falling within the first and third quadrants mainly exhibited similar trends in gene expression changes for both KOvsWT and HFDvsND conditions, while those in the other quadrants showed opposite changes. The genes were further categorized into five types, including “Same” (KOvsWT and HFDvsND exhibited the same trends in gene expression changes), “NotSig” (not statistically significant), “Diff” (KOvsWT and HFDvsND displayed opposite trends in gene expression changes), “KOvsWT Sig” (the gene expression changes were specific to KOvsWT), and “HFDvsND Sig” (the gene expression changes were specific to HFDvsND) (Fig. 3a). Simultaneously, we analyzed gene expression changes in different liver zonation regions to gain a more comprehensive understanding of key gene alterations (Supplementary Fig. S3b). We observed distinct gene expression patterns in each liver zone for KOvsWT and HFDvsND conditions (Supplementary Fig. S3b). These results suggest that the occurrence of MASH is regulated by the coordinated actions of different genes within liver zonation.

Moreover, we conducted a pathway analysis to assess the enrichment of the four different types of significantly altered genes in the KEGG pathways (Fig. 3b). The unsaturated fatty acid biosynthesis and metabolism, and fatty acid metabolism signaling pathways were notably enriched in genes exhibiting “Same” trend in their expression changes (Fig. 3b). However, we observed that pathways playing a pivotal role in lipid metabolism and related processes, such as lipid droplet formation, tissue distribution, and positioning, as well as positive regulation of various aspects of lipid metabolism, phospholipid metabolism, and lipid transport, were particularly enriched in genes displaying “Diff” trends in their expression changes (Fig. 3b). Furthermore, genes with “KOvsWT Sig” trend were primarily related to the regulation of lipid catabolism, while genes with “HFDvsND Sig” trend were associated with lipid transport and export within cells, as well as steroid hormone secretion (Fig. 3b). These regulatory mechanisms collectively maintain the balance and stability of lipid metabolism in *Gpr35* KO mouse liver.

Considering the significance of gene expression patterns in spatial characterization and MASLD, we took three factors highly associated with lipid droplet formation, lipid storage, and fatty acid biosynthesis—cell death-inducing DNA fragmentation factor alpha (DFFA)-like effector A (*Cidea*), peroxisome proliferator-activated receptor gamma (*Pparg*), and acetyl-CoA carboxylase alpha (*Acaca*)—and mapped their expression into pathological tissues. The expression trends of these genes remained consistent with the patterns observed in the two distinct conditions mentioned above (Fig. 3c). These discoveries offer valuable insights into the gene expression alterations linked to the presence or absence of GPR35 and different dietary conditions, providing a clearer understanding of the potential role of GPR35 in the progression of MASLD and MASH.

### Spatial metabolite features in liver zonation under different conditions

**Figure 4:**
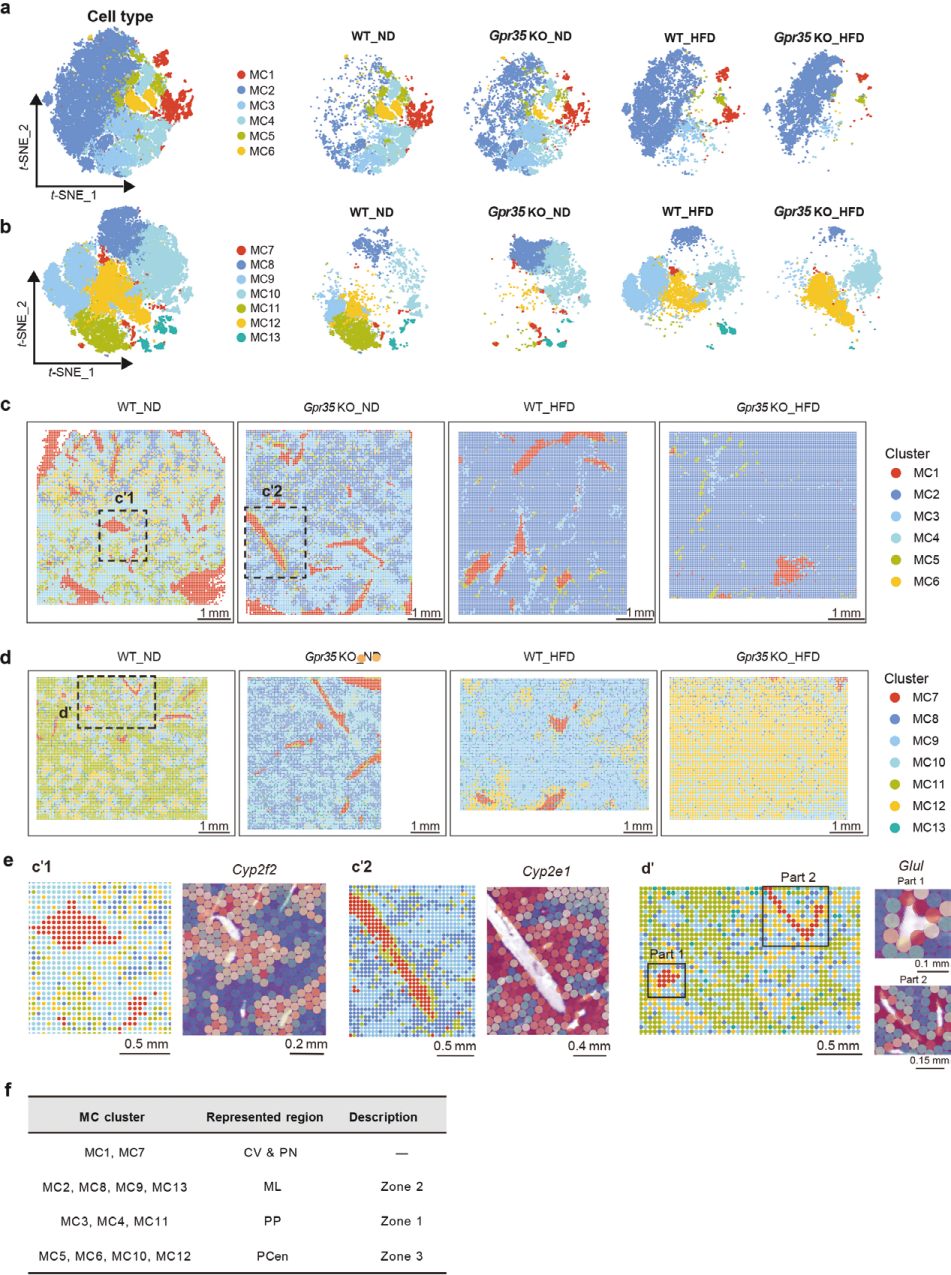
Spatial metabolite features in WT and *Gpr35* KO mouse liver zonation under ND and HFD conditions. (a and b) *t*-SNE plot of SM spots colored by the final MCs determined by negative (a) and positive (b) ion mode of MALDI-MS. (c and d) Spatial distribution of SM profile-based clusters in WT and *Gpr35* KO mouse livers under ND and HFD conditions in both negative (c) and positive (d) ion modes of MALDI-MS. The clusters are marked by different colors and named MCs. (e) Representative magnified images from c (cʹ1: WT_ND, cʹ2: *Gpr35* KO_ND, negative mode) and d (dʹ: WT_ND, positive mode), and spatial distributions of the PN zonation marker gene *Cyp2f2* and CV zonation marker genes *Glul* and *Cyp2e1* in the corresponding regions of ST. (f) The summary of the alignment between the MC clusters and liver zonation.

To comprehensively explore the distribution of metabolites regulated by GPR35 in the mouse liver under MASLD conditions, we conducted spatially resolved (25–50 μm^2^ pixel size) metabolite and lipid analyses using matrix-assisted laser desorption/ionization Fourier transform ion cyclotron resonance (MALDI-FTICR) imaging. In the negative and positive ion modes, we obtained an average of 10,632 and 10,105 metabolite points per sample, respectively, with the majority of these metabolites being phospholipids. The samples were analyzed in both ion modes and then subjected to clustering to group them into several initial clusters (Supplementary Fig. S4a and b; see details in the Methods). By assessing the similarity among these initial clusters, as evidenced by the correlation distance between clusters (Supplementary Fig. S4c), we identified six clusters in negative ion mode (labeled as metabolite clusters (MCs), MC1−MC6) (Fig. 4a; Supplementary Fig. S4d) and seven clusters in positive ion mode (labeled as MC7−MC13) (Fig. 4b; Supplementary Fig. S4e). Among these clusters, MC1 and MC7 represented the CV and PN regions, while MC2, MC8, MC9, and MC13 were corresponding to ML (Zone 2) (Fig. 4c–f). MC3, MC4, and MC11 clusters represented the PP (Zone 1), which was closer to the PV and slightly farther from the CV (Fig. 4c–f). MC5, MC6, MC10, and MC12 clusters represented the PCen (Zone 3), primarily located near the CV (Fig. 4c–f). The distribution of MCs also aligned closely with the liver histological structure (Fig. 4c and d). More importantly, the content and distribution of MCs were different between each liver sample (Fig. 4a−d, f). Notably, the distinctive patterns of cluster distributions were particularly pronounced in negative ion mode (Fig. 4c), for instance, MC3, MC4, and MC6 significantly reduced their proportion in *Gpr35* KO and HFD conditions, while MC2 increased. Comparing the clusters with spatial transcriptome profiles, we also noted that the dysregulated metabolic status within liver zonation and its spatial structure under HFD conditions remained consistent with previous reports [23]. To further enhance the interpretability of these clusters, we overlaid the metabolite-driven profiles with the spatial distribution of well-established marker genes. This integration enabled us to categorize the samples into more readily interpretable clusters (Fig. 4e; Supplementary Fig. S5). These findings indicated the presence of unique MCs that are specifically distributed within liver zonation under different conditions, which requires in-depth investigation.

### Spatial distribution of phospholipids in mouse liver under MASLD conditions

**Figure 5:**
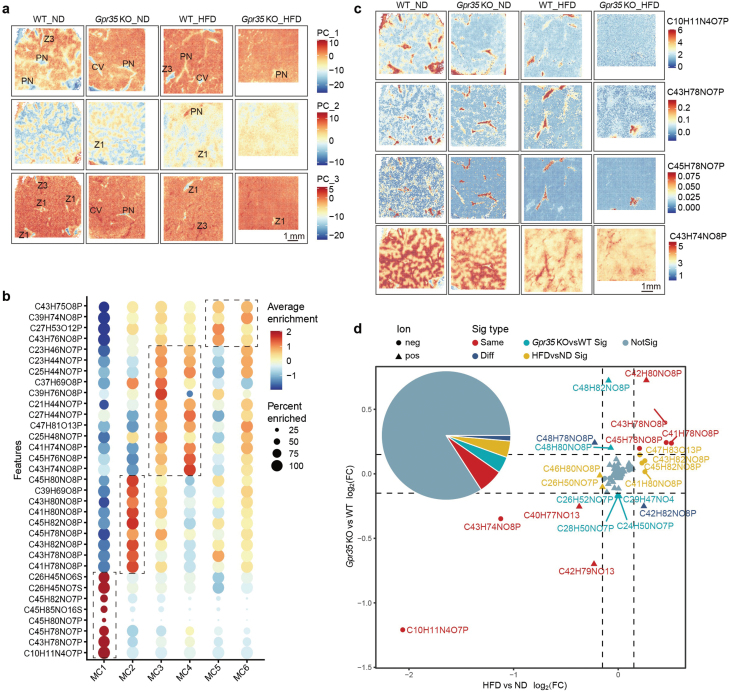
Spatial distribution of phospholipids in WT and *Gpr35* KO mouse livers under ND and HFD conditions. (a) Spatial distributions of the first three PCs (PC1, PC2, and PC3) determined by the negative ion mode in the four different liver tissue samples. Representative liver histological structures were annotated manually. V: certain vein; PN: portal nodes; Z1: zone 1; Z3: zone 3. (b) Dot plot of the relative intensity of metabolites. The colors and point sizes represent the average enrichment and enriched percentage of specific phospholipids in each cluster. (c) Spatial distribution of C10H11N4O7P, C43H78NO7P, C45H78NO7P, and C43H74NO8P in WT and *Gpr35* KO mouse livers under ND and HFD conditions. These metabolites are specifically distributed in CV, PN, and Zone 1 or Zone 3 areas, respectively. (d) Scatter plot of the differential enrichment of phospholipids in two different comparison manners. The X-axis represents the average log_2_(FC) (avg_log_2_(FC)) computed by comparing the HFD and ND liver tissues (HFDvsND). The Y-axis represents the avg_log_2_(FC) computed by comparing the *Gpr35* KO and WT liver tissues (KOvsWT). The point colors represent the significance type.

According to the principal component analysis (PCA) results, the profiles of the first several principal components (PCs, PC1−PC3) showed strong correlations with the liver zonation. PC1 demonstrated a positive association with cluster MC2 (ML or Zone2) and cluster MC1 (CV and PN), while exhibiting negative associations in both PC1 and PC3 (Fig. 5a). The differences in negative loadings between PC1 and PC3 were not identical, indicating subtle variations in metabolites depending on more refined area classification. The MC3 and MC4 clusters (PP) can be distinguished based on their lipid profiles and subsequent PCA scores, with negative loadings in the PC2 (Fig. 5a). Similarly, the positive loading of PC2 represented the MC11 cluster (PP) in the positive ion mode (Supplementary Fig. S6a). It appeared that the PP was more prominently distinguished in the PCA.

Next, we aimed to elucidate the distinctive features of representative metabolites within different MCs in liver zonation under varying conditions. Notably, we observed that metabolites such as C10H11N4O7P (3ʹ,5ʹ-cyclic inosine monophosphate (IMP), a widely recognized purine 3ʹ,5ʹ-cyclic nucleotide with critical roles as a secondary messenger in hormone action and cellular signal transduction [24]), C43H78NO7P (phosphatidylethanolamine (PE) O-38:5 or P-38:4), and C45H78NO7P (PE O-40:7 or P-40:6) were significantly enriched in the CV and PN (MC1 cluster) (Fig. 5b). Notably, C10H11N4O7P exhibited a noticeable decrease under HFD conditions and had a different distribution compared to metabolites C43H78NO7P and C45H78NO7P (Fig. 5c). Considering the proximity of these metabolites, it can be inferred that C10H11N4O7P represented the CV and PN regions in ND conditions, however, under HFD conditions, C10H11N4O7P only represented the CV, while C43H78NO7P and C45H78NO7P represented the PN region (Fig. 5c). Additionally, metabolites such as C43H74NO8P (PE (38:6) or phosphatidylcholine (35:6)) and C45H76NO8P (PE (40:7) or phosphatidylcholine (37:7)) exhibited significant variations within the MC4 cluster, distinguishing them from other clusters (Fig. 5b and c). In addition, we also identified distinct MCs in different liver zones by positive ion mode, for instance, C47H93N2O6P (+H) (Sphingomyelin (d 42:2)), C40H80NO8P (+H) (phosphatidylcholine (32:0)), and C40H80NO8P (+Na) (PE (35:0)) were specifically enriched in the MC1 cluster (CV and PN) and C44H82NO8P (+Na) (phosphatidylcholine (36:3)) and C42H82NO8P (+Na) (phosphatidylcholine (34:1)) were only enriched in the MC13 cluster (ML, Zone 2) (Supplementary Fig. S6b). Furthermore, MC11, representing the PP, exhibited high expression of polyunsaturated phosphatidylcholine compounds, including C26H50NO7P (+Na) (phosphatidylcholine (18:2) or lyso-phosphatidylcholine (18:2)), C24H50NO7P (+Na) (phosphatidylcholine (16:0) or lyso-phosphatidylcholine (16:0)), C30H50NO7P (+Na) (phosphatidylcholine (22:6) or lyso-phosphatidylcholine (22:6)), and C28H50NO7P (+Na) (phosphatidylcholine (20:4) or lyso-phosphatidylcholine (20:4)) (Supplementary Fig. S6c). These findings suggest that the mentioned metabolites have the potential to serve as marker metabolites for the PP.

To investigate the key metabolites specifically involved in MASLD, we conducted a comprehensive analysis of the metabolite profiles under HFD conditions in *Gpr35* KO mice (Fig. 5d). Our findings revealed that the metabolite C10H11N4O7P (3ʹ,5ʹ-cyclic IMP) exhibited a significant decrease in both the KOvsWT and HFDvsND conditions (Fig. 5d). It is noteworthy that this metabolite was specifically associated with the CV and PN regions (Fig. 5b and c). However, metabolite associated with the PP (Zone 1), C42H80NO8P (phosphatidylcholine (34:2) or PE (37:2)), and those linked to the ML (Zone 2), C41H78NO8P (PE (36:2) or phosphatidylcholine (33:2)) and C43H78NO8P ((PE (38:4) or phosphatidylcholine (35:4)), showed upregulation in both the KOvsWT and HFDvsND conditions (Fig. 5d). Furthermore, several metabolites exhibited enrichment depending on *Gpr35* expression in different liver zonation, for instance, C48H82NO8P (phosphatidylcholine (40:7), linked to Zone 3) and C26H50NO7P (phosphatidylcholine (18:2) or lyso-phosphatidylcholine (18:2), linked to Zone 1) (Fig. 5d). On the other hand, metabolites like C46H80NO8P (phosphatidylcholine (38:6), linked to Zone 1) and C41H80NO8P (PE (36:1) or phosphatidylcholine (33:1), linked to Zone 2) showed enrichment dependent on the dietary conditions (Fig. 5d). These results suggest that alterations in metabolites contribute to the modulation of liver zonation under HFD conditions.

### Spatial molecular signatures influenced by GPR35 in liver zonation under HFD

**Figure 6:**
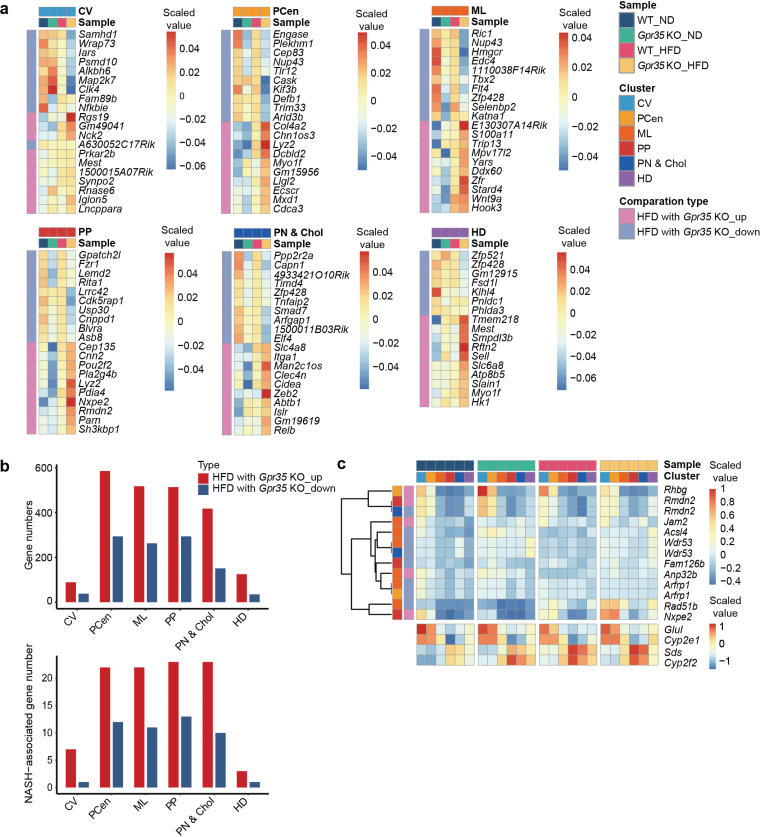
Spatial molecular signatures influenced by GPR35 in liver zonation under HFD conditions. (a) Heatmaps displaying the gene expression patterns within different liver zonation clusters in WT and *Gpr35* KO mouse livers under both ND and HFD conditions. Only the top 10 upregulated and downregulated genes influenced by both the HFD and *Gpr35* KO conditions for each liver zonation cluster are shown. The terms “HFDwithGpr35_up” and “HFDwithGpr35_down” refer to genes whose expressions are changed when there are alterations in both HFD and *Gpr35* KO conditions. The terms “up” and “down” refer to the directional trends of gene changes when conditions are altered, with associations to either HFD or *Gpr35* KO. (b) Bar plot of the numbers of DEGs (upper panel) and genes associated with NASH (nonalcoholic steatohepatitis) (lower panel) affected by both the HFD and *Gpr35* KO conditions in each liver zonation cluster. “Genes associated with NASH” are those that exhibit differential expression (Wilcox test, Benjamini and Hochberg correction method, adjusted *P* value < 0.05) in either the “HFDwithGpr35_up”” or “HFDwithGpr35_down” conditions, as well as being present in the DisGeNET. (c) Heatmap displaying the gene expression patterns associated with *Gpr35* KO and HFD within different liver zonation clusters in WT and *Gpr35* KO mouse livers both under ND and HFD conditions. The expressions of classical spatial marker genes *Glul*, *Cyp2e1*, *Sds*, and *Cyp2f2* were also displayed.

To gain further insights into the spatial molecular signatures influenced by GPR35 in liver zonation under HFD conditions, we investigated spatial gene expression data in specific liver zones under HFD conditions (Fig. 6a and b). Interestingly, we observed that the number of upregulated genes consistently exceeded the number of downregulated genes across all clusters in the presence of GPR35 and HFD conditions (Fig. 6b, upper panel). Moreover, there was an increased number of MASH-associated genes under the influence of both GPR35 and HFD conditions (Fig. 6b, lower panel). These findings suggest a prevailing trend of gene upregulation in response to the combined influence of GPR35 and HFD conditions in the analyzed clusters.

Next, we observed alterations in the expression of several genes related to anti-inflammation, lipogenesis, and the MASH process in different liver zones of *Gpr35* KO mice under HFD conditions, including *S100a11* [25], *Smad7* [26], *Zeb2* [27], *Smpdl3b* [28], *Hmgcr* [29], *Nck2* [30], *Hk1* [31], *Usp30* [32], E74 like E-twenty six (ETS) transcription factor 4 (*Elf4*) [33, 34] and *Cidea* [35] (Fig. 6a). Furthermore, a MASH-associated gene, steroidogenic acute regulatory protein (StAR)-related lipid transfer domain protein 4 (*Stard4*), was found to be upregulated in the liver zone 2 (ML) of *Gpr35* KO mice under HFD conditions (Fig. 6a). These results suggest that the interaction between GPR35 and the aforementioned genes plays a crucial role in providing protective effects against MASH in the context of an HFD.

Subsequently, we explored the alteration trends of all GPR35 and HFD-associated genes across various samples, revealing significant changes in at least one sample within the liver zonation clusters (Fig. 6c). It is worth mentioning that previous research has suggested a targeting gene acyl-CoA synthetase long-chain family member 4 (*Acsl4*), which could potentially offer an alternative therapeutic approach for MASLD [36].

### CIDEA and ELF4 are potential downstream regulators of GPR35 in MASLD

**Figure 7:**
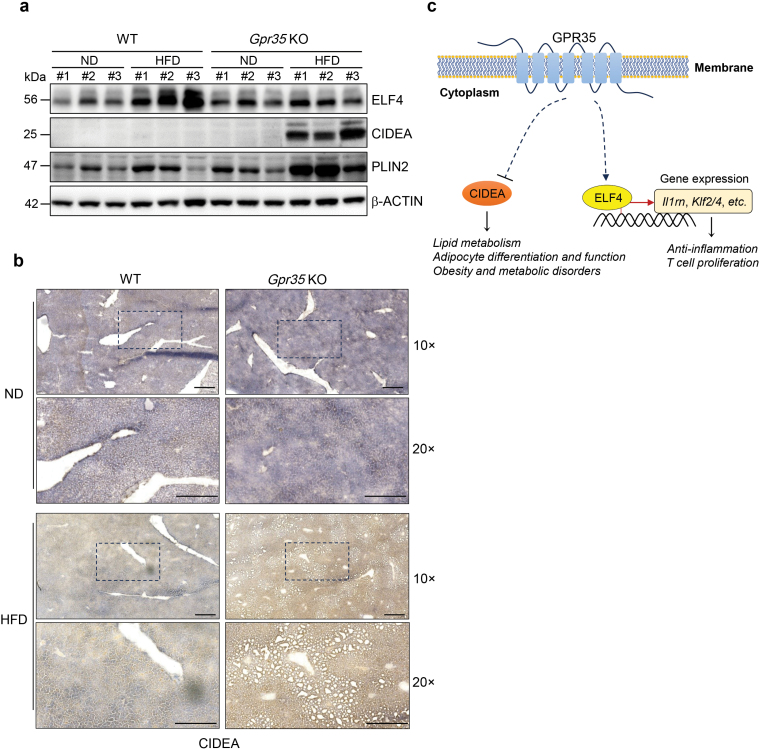
CIDEA and ELF4 are potential downstream regulators of GPR35 in MASLD. (a) Immunoblotting analysis of indicated antibodies in WT or *Gpr35* KO mouse liver tissues under ND or HFD conditions. (b) Representative IHC staining of CIDEA in WT or *Gpr35* KO mouse liver tissues under ND or HFD conditions. Scale bars, 100 μm. (c) Diagram of discoveries in this study.

To validate our findings, we assessed the expression levels of the anti-inflammation factor ELF4 and key lipogenesis factor CIDEA in mouse livers under both ND and HFD feeding conditions [33–35]. We observed a significant upregulation of ELF4 expression in WT mouse livers under HFD feeding conditions, whereas this upregulation was inhibited in *Gpr35* KO mouse livers (Fig.7a; Supplementary Fig. S7a and b). Immunohistochemical (IHC) staining further confirmed the decreased expression of ELF4 in *Gpr35* KO mouse livers compared to WT mouse livers under HFD feeding conditions (Supplementary Fig. S7b). These findings indicated a more severe liver inflammation in *Gpr35* KO mice. Conversely, the expression of CIDEA significantly increased in *Gpr35* KO mouse livers compared to WT mouse livers under HFD feeding conditions (Fig.7a and b; Supplementary Fig. S7a). Additionally, we examined the key lipid droplet-associated protein perilipin-2 (PLIN2) [37] in mouse livers under both ND and HFD feeding conditions and found that PLIN2 was significantly more abundant in *Gpr35* KO mouse livers compared to WT mouse livers (Fig. 7a). In parallel, we also observed that *Gpr35* KO mouse livers accumulated more lipid droplets than WT mouse livers under HFD feeding conditions (Fig. 7b; Supplementary Fig. S7b). These results suggest that GPR35 plays a significant role in lipid generation and the progression of MASLD.

## Discussion

In recent years, remarkable progress has been achieved in unraveling the intricacies of liver zonation due to the advancements in single-cell profiling and spatial omics technologies. These breakthroughs have revolutionized our comprehension of liver cell organization and provided invaluable insights into gene expression and metabolite distribution, particularly in the context of pathologies like MASLD and MASH. However, despite these technological strides, comprehensive investigations into the functions of disease-associated genes in MASLD using state-of-the-art methods have been relatively limited. In this study, we capitalized on these cutting-edge techniques and accumulated knowledge to illuminate the pivotal role of the *Gpr35* gene in liver zonation and its profound impact on the spatial distribution of genes and metabolites. By thoroughly characterizing the effects of GPR35, our research provides valuable insights into its function within the realm of MASLD (Fig. 7c). This, in turn, contributes significantly to our deeper understanding of the underlying mechanisms that propel MASLD and sheds light on the specific role of *Gpr35*.

GPR35 has been implicated in playing a role in lipid metabolism and is a promising therapeutic target for lipid metabolism disorder-related diseases, like MASLD. Recently, GPR35 was found to regulate cholesterol homeostasis in high-fat/cholesterol/fructose (HFCF)-dieted mouse model by inducing STARD4 expression [9]. Interestingly, we found that with only an HFD, without high fructose, the MALSD-like phenotypes can be induced in *Gpr35* KO mice, underscoring the pivotal role of GPR35 in modulating hepatic metabolic disruptions. Our study suggested that GPR35 also plays a very significant role in lipid storage and lipid droplet formation in addition to cholesterol homeostasis regulation. Furthermore, sphingolipid metabolites like ceramides and sphingomyelin are also regulated by GPR35, implying their connection with the inflammation response in MASLD and MASH [38–40]. We also revealed that each zone within the hepatic lobule possesses a distinct metabolic state and function, leading to notable variations in both metabolites and gene transcription (Figs 2 and 5; Supplementary Fig. S7c), such as sphingolipid metabolism, which is thriving in the periportal zone. Interestingly, we have observed a region exhibiting high expression of injury response-related *Saa1/2/3* genes, and this expression is closely linked to the absence of *Gpr35* and the presence of an HFD. These findings strongly suggested that *Gpr35* deficiency impairs the repair mechanisms for hepatocyte damage, ultimately contributing to the exacerbation of MASLD. Taken together, our study underscores the zonal specificity of GPR35 in regulating liver metabolism and its capacity to modulate specific metabolic pathways in distinct liver areas.

The identification of specific genes regulated by GPR35 in the hepatic steatosis process carries substantial significance, as it offers valuable insights into the role of GPR35 and unveils potential targets for treating fatty liver. Among these genes, several have been previously linked to the development of fatty liver, including *Acaca*, *Fasn*, *Pparγ*, *Cidea*, cardiotrophin-like cytokine factor 1 (*Clcf1*), and *Acly*. Notably, in HFD conditions, *Gpr35* deficiency led to the upregulation of *Cidea*, which correlated with exacerbated hepatic steatosis in *Gpr35* KO mice. CIDEA is known to facilitate the fusion of lipid droplets into larger structures within mammalian cells through a phosphatidic acid-binding amphipathic helix [41, 42]. Dysregulation of CIDEA-mediated lipid droplets has been associated with MASH [43], suggesting the role of GPR35 in maintaining tightly regulated lipid droplet homeostasis, likely through the control of downstream CIDEA. Another intriguing discovery is the relationship between *Gpr35* and *Stard4*, which consisted of recent research highlighting that GPR35 prevents MASH through STARD4-mediated hepatocyte cholesterol homeostasis [9]. Additionally, our study has unveiled novel associations between GPR35 and the anti-inflammation factor ELF4, which have not been extensively explored in the context of MASH progression. Further investigation of the relation between ELF4 and sphingolipid metabolism in fatty liver would be worthwhile. These findings provide a valuable reference for delving into new mechanisms in MASLD and may pave the way for innovative therapeutic approaches.

Our SM study utilizing matrix-assisted laser desorption/ionization mass spectrometry imaging (MALDI-MSI) has discovered several new makers for the identification of the histological features in the liver, and also provided valuable insights into the role of GPR35 in MASLD. The distinctive features of representative metabolites within different metabolism clusters were examined to gain a deeper understanding of their distribution in different regions of the liver. Notably, we have identified 3ʹ,5ʹ-cyclic IMP (C10H11N4O7P) as a marker for the CV and portal vein region, known for its noncanonical cyclic nucleotide nature, associated with Rho kinase activity, and recognized as a second messenger in vascular wall [44]. This finding suggests the potential utility of 3ʹ,5ʹ-cyclic IMP as a specific marker for identifying blood vessels in liver biopsies in future studies. Furthermore, the alteration in 3ʹ,5ʹ-cyclic IMP implies that GPR35, a Rho kinase activator, may rely on 3ʹ,5ʹ-cyclic IMP-mediated signal transduction and play a protective role in maintaining vessel integrity [5, 6]. Moreover, C43H74NO8P (PE (38:6)) has emerged as a promising marker metabolite for the PP. In the positive ion mode, cluster MC11, also representing the PP, exhibited an enrichment of polyunsaturated PC compounds. These findings provide valuable insights into the spatial distribution of specific metabolites and their potential associations with different liver zones.

Upon further exploration of metabolite alterations, we uncovered several marker metabolites associated with GPR35 and diet. Notably, C48H82NO8P (PC (40:7)) emerged as a marker for the PCen, while C26H50NO7P (phosphatidylcholine (18:2) or lyso-phosphatidylcholine (18:2)) was identified as a marker for the PP under HFD conditions. More importantly, C48H82NO8P (phosphatidylcholine (40:7), linked to Zone 3) was recognized as a marker for the PCen, closely associated with the function of GPR35. These findings provide essential insights into the metabolic shifts associated with GPR35 and its interplay with dietary conditions.

The distinctive metabolic clusters and their spatial distribution shed light on the zonal heterogeneity of liver metabolism and potential GPR35-mediated regulatory mechanisms. Our findings enhance our understanding of how GPR35 influences metabolite and lipid profiles in different liver regions and open new avenues for investigating the intricate molecular mechanisms underlying MASLD pathogenesis. In conclusion, our research delves into the intricate relationship between GPR35, liver zonation, and MASLD pathogenesis. By utilizing cutting-edge techniques, we have uncovered important gene and metabolite associations that shed light on the role of GPR35 in regulating liver metabolism. These findings have significant implications for understanding MASLD and identifying potential therapeutic targets for the treatment of fatty liver disease. Future investigations in this area may pave the way for novel interventions and improved management of MASLD.

## Materials and methods

### Generation of WT and *Gpr35* KO mice

WT mice and *Gpr35*-deficient (*Gpr35* KO) mice with C57BL/6 genetic background were purchased from BIORAY LABORATORIES Inc. (Shanghai, China). *Gpr35* KO mice were bred with C57BL/6 mice to obtain *Gpr35* heterozygotes. *Gpr35* KO mice and littermate controls obtained from heterozygote crosses were used for all experiments. All institutional and national guidelines for the care and use of laboratory animals were followed. All animal care and experimental procedures were approved by the Research Ethics Committee of Dalian Institute of Chemical Physics, Chinese Academy of Sciences (No. DICPEC2314).

### HFD model

*Gpr35* KO mice were utilized in this research to establish an MASLD model. The mice were divided into four groups: WT mice on an ND, *Gpr35* KO mice on an ND, WT mice on an HFD, and *Gpr35* KO mice on an HFD. ND was consisted of standard rodent chow, while HFD (XTHF60-1) was purchased from Jiangsu Xietong Pharmaceutical Bio-engineering Co., Ltd. (Nanjing, China), which contained 60 % of fat. The experimental duration was 14 weeks for all groups. During the experimental period, the mice were housed in a controlled environment with a 12-h light/12-h dark cycle and provided *ad libitum* access to food and water. Body weight measurements were recorded every two weeks for both the control and experimental groups throughout the 14-week intervention. At the end of the 14-week HFD intervention, the mice were euthanized, and tissues of interest were collected for further analysis. iWAT, eWAT, and rWAT were carefully dissected, and their weight was recorded.

### IPGTT

Following the 14-week HFD intervention, an IPGTT was conducted to assess glucose tolerance in the mice. Prior to the test, mice were fasted for 12 h to ensure accurate baseline measurements. Subsequently, mice were administered intraperitoneally with 0.2 g/mL d-glucose solution (2 g/kg body weight), and blood samples were collected at regular intervals, including 0, 30, 60, and 120 min after glucose administration. Glucose levels in the collected blood samples were measured using a reliable and calibrated glucometer.

### Metabolite extraction from liver samples

Sheared liver tissues were weighed and 500 μL methanol with internal standards (ISs, including carnitine C16:0-d3, ceramide d18:1-d7/15:0, lyso-phosphatidylcholine 26:0-d4, phosphatidylcholine 16:0/18:1-d31, triacylglycerol (TAG) 16:0/18:0/16:0-d5, PE 16:0/18:1-d31, sphingomyelin d18:1/15:0-d9, and free fatty acid (FFA) 16:0-d3) was added. All ISs with a final concentration of 0.4 μg/mL were added. A grinding apparatus was used for homogenization (1000 rpm, 1 min) followed by the addition of 500 μL chloroform and vortex for 30 s. After phase breaking using 200 μL water and centrifugation (13,000 *g*, 4°C, 15 min), 320 μL organic phase was collected and freeze-dried for LC–MS/MS analysis. Quality control (QC) sample was also prepared by combining the organic phase and then vacuum dried to evaluate the analytical quality.

### Lipidomics analysis

Lipidomics analysis was performed by a 1290-6546 LC/Q-TOF mass spectrometer (Agilent Technologies). Lyophilized samples were reconstituted in a resolve reagent (dichloromethane:methanol = 2:1). After intense vortex mixing, a dilution reagent (isopropanol:methanol = 2:1, containing 5 mmol/L ammonium acetate) with three times the volume of the resolve reagent was added. Chromatographic separation was performed on an UPLC column (ACQUITY UPLC® BEH C8 1.7 μm, 2.1 mm × 100 mm, Waters) with the following gradient: 50% A for 0−1.5 min; 50%−15% A for 1.5−9 min; 100% B for 9−11 min; 50% A for 11−13 min. Mobile phase A was 60% acetonitrile and 40% water, containing 10 mmol/L ammonium acetate. Mobile phase B was 90% isopropanol and 10% acetonitrile, with 10 mmol/L ammonium acetate. The flow rate was set to 0.3 mL/min and the injection volume was 5 µL. The column temperature and the multisampler temperature were set to 60°C and 10°C, respectively. The MS was in high-resolution mode. Gas temperature was 320°C with the sheath gas temperature at 350°C. The acquisition rate was set to 9261 transients/spectrum and both the negative and the positive mode was used, the *m*/*z* scan range was 50–1500 Dalton, and the fragmentor was 175V. For MS/MS, both 15 eV and 30 eV were used as collision energy. MassHunter Qualitative Analysis 10.0 (Agilent Technologies) was used for chromatogram review and IS peak area integration. The processed data were exported for further analysis. Samples were randomized concerning run order to avoid batch effects. Additionally, the QC samples were identically inserted into the analytical sequence to monitor the reproducibility of the analytical method.

Mass Spectrometry-Data Independent Analysis software (MS-DIAL) was used to compare and process data. A total of 199 lipid metabolites were identified by selecting species that matched both MS (≤ 5 ppm) and MS/MS spectra and were detected in all samples. After manually integrating the peak area of metabolites, they were divided by the peak area of the same type of IS and tissue mass in the sample to obtain the relative concentration.

### ST sequencing method

Livers from four groups of mice, namely WT_ND, *Gpr35* KO_ND, WT_HFD, and *Gpr35* KO_HFD, were collected and embedded in optimal cutting temperature compound (OCT), and were frozen on dry ice immediately after the mice were sacrificed. Sample preparation, sectioning, staining, imaging, library preparation, and sequencing were performed following the Visium protocol (10× Genomics).

### Spatial transcriptome data analysis

The Space Ranger software was utilized to align the ST data with the corresponding H&E image and the reference genome sequence (GRCm38). To ensure data quality, spots (locations) with a feature number (nFeature_Spatial) below 250 or percent of mitochondrial genes (percent.mito) higher than 15% were removed. After quality control, 3807 high-quality (HQ) spots were obtained for WT_ND, 3565 HQ spots for *Gpr35* KO_ND, 4517 HQ spots for WT_HFD, and 4489 HQ spots for *Gpr35* KO_HFD, respectively. Additionally, all mitochondrial genes and ribosomal genes were excluded from the analysis to eliminate potential interference from these genes during downstream analysis.

The analysis pipeline used in this study was based on the Seurat package (v 4.4.0) [45]. All samples were firstly processed by SCTransform, and subsequently merged using the SelectIntegrationFeatures, PrepSCTIntegration, FindIntegrationAnchors, and IntegrateData functions, employing normalization with the method set to “SCT”. Subsequently, PCA, *t*-SNE plots, and clustering were generated using the RunPCA, RunTSNE, FindNeighbors, and FindClusters functions. The spatial gene distribution and the spatial adjacency relationship between clusters were visualized using the SpatialFeaturePlot function. To identify genes significantly influenced by specific experimental conditions or biological factors between different sample groups, the FindAllMarkers and FindMarkers functions were used.

In this study, the SPATA2 software package (version 2.0.4) and the Seurat tool (version 5.0.2) were utilized to plot the spatial trajectory of genes, demonstrating the changing trends of characteristic marker genes from PN to CV [46].

Two different forms of pathway enrichment analysis were utilized to gain insight into the biological functions of the spatial spots and identified clusters respectively. The gene set variation analysis (GSVA) was performed to assess the relative enrichment of gene sets across each spatial spot using a nonparametric approach. On the other hand, over-representation concerning the differentially expressed genes (DEGs) or cluster markers involved in each pathway was examined based on the hypergeometric distribution. The pathway information was obtained from the KEGG database, while the biological process (BP) terms for the four different types of DEGs were from the Gene Ontology (GO) database. The GSVA method was performed based on the GSVA package (v1.46.0). The over-representation analysis was performed by the compareCluster function from the clusterProfiler package (v4.7.1.3) [47].

### Tissue preparation and matrix deposition for MALDI imaging

The adjacent slices (of 20 μm) from that for ST were pasted on an ITO-coated glass slide for analysis by MALDI-MSI. After 10 min of desiccation, the surrounding OCT was peeled off, briefly washed with PBS, and subjected to matrix deposition. The matrices of 1,5-diaminonaphthalene (1,5-DAN) were evenly coated on the sample surface using a sublimation device. The matrix holder was filled with approximately 300 mg 1,5-DAN, the matrix power was then heated to 170°C, and the vapor covered the specimen surface for 14 min. After sublimation, the samples were rehydrated at −20°C overnight and ready for MALDI-MS after a brief desiccation.

### MALDI imaging

Mass spectra were acquired on a SolariX 15.0T FT-ICR MS (Bruker, Daltonics) equipped with a dual ion source (ESI and MALDI) and a Smartbeam II laser (2 kHz) operated in both positive ion and negative ion mode. The size was set to 1M, the mass range was set to *m/z* 150–1800, and a time of flight of 1.0 ms were used. Prior to analysis, the acquiring method was externally calibrated using NaTFA in linear mode first, and then calibrated online using a series of reference ions within the imaging at a 25 μm or 50 μm spatial resolution, in which laser power of 80%, frequency of 1000 Hz, and laser focus of small were utilized. Spectra produced by 30 laser shots in positive mode or 80 laser shots in negative mode were summed, averaged, and recorded. In positive mode, *m/z* 314.1526 (C20H18N4+), 758.5694 (C42H81NO8P+), and 782.5670 (C42H82NO8PNa+) were used as internal reference ions and 3,000,000 were set as the absolute threshold, while in negative mode, *m/z* 312.1380 (C20H15N4−), 742.5392 (C41H77NO8P−), 762.5079 (C43H73NO8P−), 766.5392 (C43H77NO8P−), 790.5392 (C45H77NO8P−), and 885.5498 (C47H82O13P−) were selected as internal calibrator and 5,000,000 were set as the absolute threshold. The FT-ICR MS was controlled by ftmsControl (V 2.2.0) and fleximaging (V 2.0), and the data was analyzed using DataAnalysis (V 5.0) and flexImaging (V 2.0, Bruker, Daltonics). All samples in both the negative and positive ion mode were processed within a single day.

### SM data analysis

The obtained RAW data from the mass spectrum imaging technology were converted into .ibd and .imzML formats using the Bruker software fleximaging. Metabolites were annotated by the METASPACE annotation platform [48], and four databases were used, namely CoreMetabolome, HMDB (Human Metabolome Database), LipidMaps, and SwissLipids. Following the metabolite annotation, the original intensity data were normalized for each sample using the root mean square (RMS) method. The following analysis pipeline was based on the Seurat package (v 4.4.0) and data obtained under negative and positive ion modes were calculated separately. To integrate samples under a specific ion mode from different experimental conditions into a unified analysis, the SelectIntegrationFeatures, FindIntegrationAnchors, and IntegrateData functions were utilized, applying normalization with the method set to “LogNormalize”. Subsequently, PCA, *t*-SNE plots, and clustering were generated using the RunPCA, RunTSNE, FindNeighbors, and FindClusters function, and initial clusters were defined. Then, hierarchical clustering, utilizing the Euclidean distance between these initial clusters based on the average expression levels of metabolites that were detected in all investigated samples, was conducted to further combine some similar clusters and determine the final MCs. The spatial metabolite distribution and the spatial adjacency relationship between clusters were also visualized using the SpatialFeaturePlot function. To identify metabolites significantly influenced by specific experimental conditions or biological factors between different sample groups, the FindAllMarkers and FindMarkers functions were used. The metabolite spatial distribution plots were generated after implementing a hot-spot removal step, which addressed the presence of individual spectra that may contain unusually high levels of analyte signal or noise. Hot-spot removal involved reducing the intensities above the 99th percentile to the 99th percentile value.

### Quantitative reverse transcriptase polymerase chain reaction (qRT-PCR) analysis

Total cellular RNA was extracted from tissue samples using RNAiso Plus from Takara Bio (Shiga, Japan) after homogenization with an IKA T10 basic Ultra-Turrax (Baden-Württemberg, Germany). The yield and purity of the isolated RNA were assessed using spectroscopic analysis, and the RNA concentration was standardized. For cDNA synthesis, the Evo M-MLV RT Kit from AG Accurate Biology Co., Ltd (Hunan, China) was employed. Target gene amplification was carried out using the SYBR Green Premix Pro Taq HS qPCR Kit from AG Accurate Biology Co., Ltd (Hunan, China) on a Bio-Rad CFX96TM Real-Time System (CA, USA). The primers used in this study were as following:

*Elf4* forward: 5ʹ-ATGCTTGCCAGCCCACTACAGA-3ʹ;

*Elf4* reverse: 5ʹ-CCATTGGTCAGCACCGTAGTCA-3ʹ);

*Cidea* forward: 5ʹ-GGTGGACACAGAGGAGTTCTTTC-3ʹ;

*Cidea* reverse: 5ʹ-CGAAGGTGACTCTGGCTATTCC-3ʹ);

*Actin* forward: 5ʹ-CATTGCTGACAGGATGCAGAAGG-3ʹ;

*Actin* reverse: 5ʹ-TGCTGGAAGGTGGACAGTGAGG-3ʹ).

The primers were designed by Origen and were purchased from Sangon Biotech Co., Ltd (Shanghai, China). The relative expression of each gene was normalized by *Actin*.

### Immuno-blotting analysis

For the immuno-blotting assay, liver tissues were homogenized with IKA T10 basic Ultra-Turrax (Baden-Württemberg, Germany) in RIPA lysis buffer (50 mmol/L Tris–HCl pH 7.4, 150 mmol/L NaCl, 0.5% deoxycholate, 1% NP40, 0.1% SDS, and 1 mmol/L EDTA) supplemented with proteinase inhibitor and phosphatase inhibitor cocktails. After denaturation, the samples were subjected to SDS–PAGE and immuno-blotting assay. Primary antibody against ELF4 was purchased from Origene Technologies, Inc. (MD, USA). Primary antibodies against ACLY, FASN, CIDEA, VINCULIN, ACTIN, and PLIN2 were purchased from Proteintech Group, Inc (IL, USA). Peroxidase-conjugated secondary antibodies were purchased from Jackson ImmunoResearch (AB_2313567 and AB_10015289).

### H&E staining

Adipose tissue and liver samples were fixed in 4% formalin and then processed for paraffin embedding. Thin sections (5 μm) were cut and subjected to H&E staining. Microscopic examination of stained sections was performed to assess the morphology and structure of adipocytes and hepatic tissues.

### Statistical analysis

All data are expressed as mean ± standard error of the mean (SEM). Statistical analyses were performed using appropriate tests, such as Student’s *t*-test or analysis of variance (ANOVA), followed by *post hoc* tests for multiple comparisons, as applicable. A *P* value < 0.05 was considered statistically significant. Statistical and computational analyses, as well as graph plotting, were conducted using R version 4.2.1. Comprehensive details, encompassing data preprocessing, data presentation, sample size, and statistical methods, were provided in respective experimental sections or figure legends. The *P* values for the expression of genes *Saa1*, *Saa2*, and *Saa3* between WT and *Gpr35* KO mice were calculated using the Wilcoxon test.

## Supplementary Material

loae021_suppl_Supplementary_Figures_S1-S8

## Data Availability

The ST data are available from GSA (accession code: CRA013704) and the SM data are available from METASPACE (Gpr35andHFD project).
