# Supplementary material for: Spatial multi-omics characterizes GPR35-relevant lipid metabolism signatures across liver zonation in MASLD
Source: Life Metab. 2024 May 31;3(6):loae021. doi: 10.1093/lifemeta/loae021 (PMC11748505; doi:10.1093/lifemeta/loae021)
Supplement: loae021_suppl_Supplementary_Figures_S1-S8 [file loae021_suppl_Supplementary_Figures_S1-S8.pdf]

Supplementary Figures

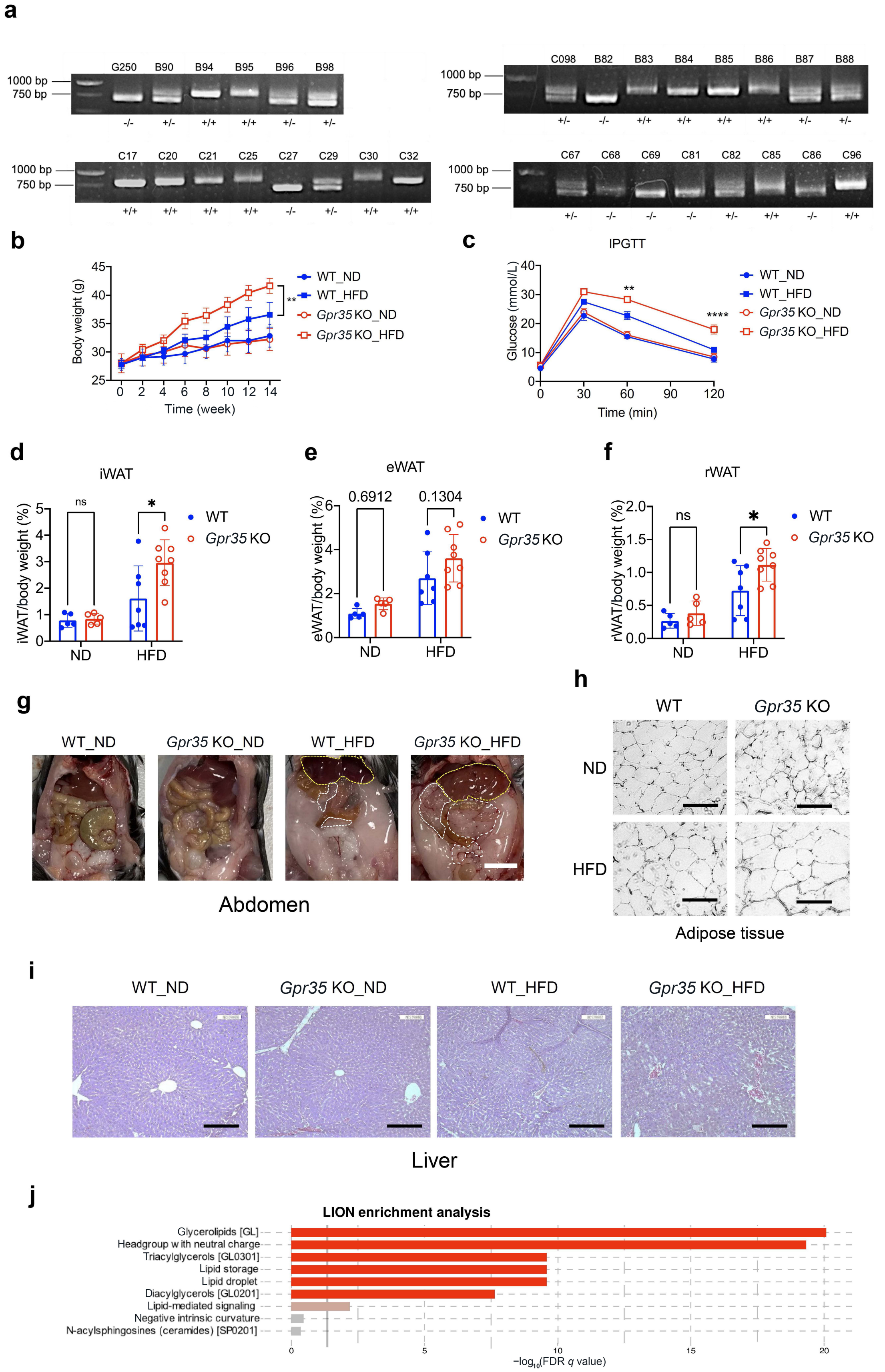

Supplementary Figure S1 *Gpr35* deficiency aggravates HFD-induced MASLD. (a) Genotyping the mice which were used in this study. (b) Body weight (n = 5). (c) Blood glucose level in IPGTT assay (n = 5). (d) Weight of inguinal white adipose tissue (n = 5 for WT_ND, n = 5 for *Gpr35* KO_ND, n = 7 for WT_HFD, n = 8 for *Gpr35* KO_HFD). (e) Weight of epididymal white adipose tissue (n = 5 for WT_ND, n = 5 for *Gpr35* KO_ND, n = 7 for WT_HFD, n = 8 for *Gpr35* KO_HFD). (f) Weight of renal white adipose tissue (n = 5 for WT_ND, n = 5 for *Gpr35* KO_ND, n = 7 for WT_HFD, n = 8 for *Gpr35* KO_HFD). (g) Representative pictures of the abdominal adipose. White dotted line: mesenteric fat; Yellow dotted line: liver. Scale bars, 1 cm. (h) H&E staining of epididymal adipose tissue. Scale bar, 100 μm. (i) H&E staining of mouse liver. Scale bar, 100 μm. (j) Lipid ontology enrichment results based on significantly changed metabolites between KO_HFD and WT_HFD samples. The analysis was performed on LION (http://www.lipidontology.com/). The X-axis shows the q- value after the $\log_{10}$ transformation, and the Y-axis shows enriched lipid ontologies.

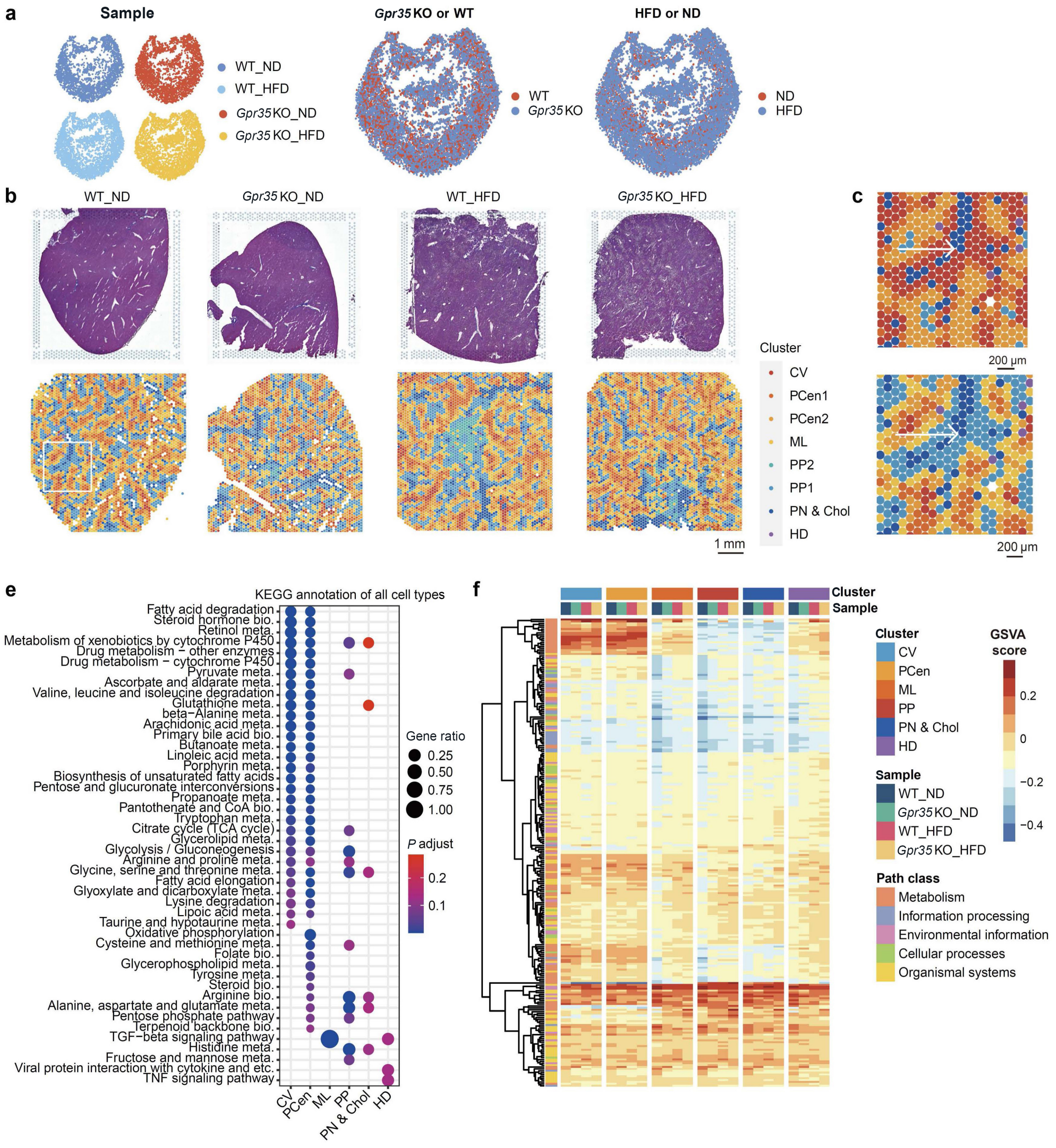

Supplementary Figure S2 Spatial mRNA features in mouse liver zonation under ND and HFD conditions. (a) *t*-SNE plot colored by sample origins. (b) Spatial distributions of the distinct liver zonation clusters (lower panel) and corresponding H&E staining images (upper panel). Different colors indicate distinct liver zonation. (c) Representative magnified image from **Fig. 2a (upper panel) and Supplementary Fig. S2a (lower panel)** WT_ND. (d) Spatial distributions of differentially expressed genes (DEGs) *Glul*, *Cyp2e1*, *Cyp2f2* and *Sds* (from left to right) in WT and *Gpr35* KO mouse livers under ND or HFD conditions (See next page). (e) KEGG pathway over-representation analysis of DEGs in each liver zonation cluster. Adjusted *P* values (Benjamini & Hochberg correction method) are indicated by colors and gene ratios are represented by point sizes. bio.: biosynthesis; meta.: metabolism. Viral protein interaction with cytokine and etc.: Viral protein interaction with cytokine and cytokine receptor. (f) KEGG-based mean GSVA enrichment score of spatial spots within each clusters in four different samples. (g) The comparison of our data with previous research under analogous sample conditions (See next page).

Supplementary Figure S2d

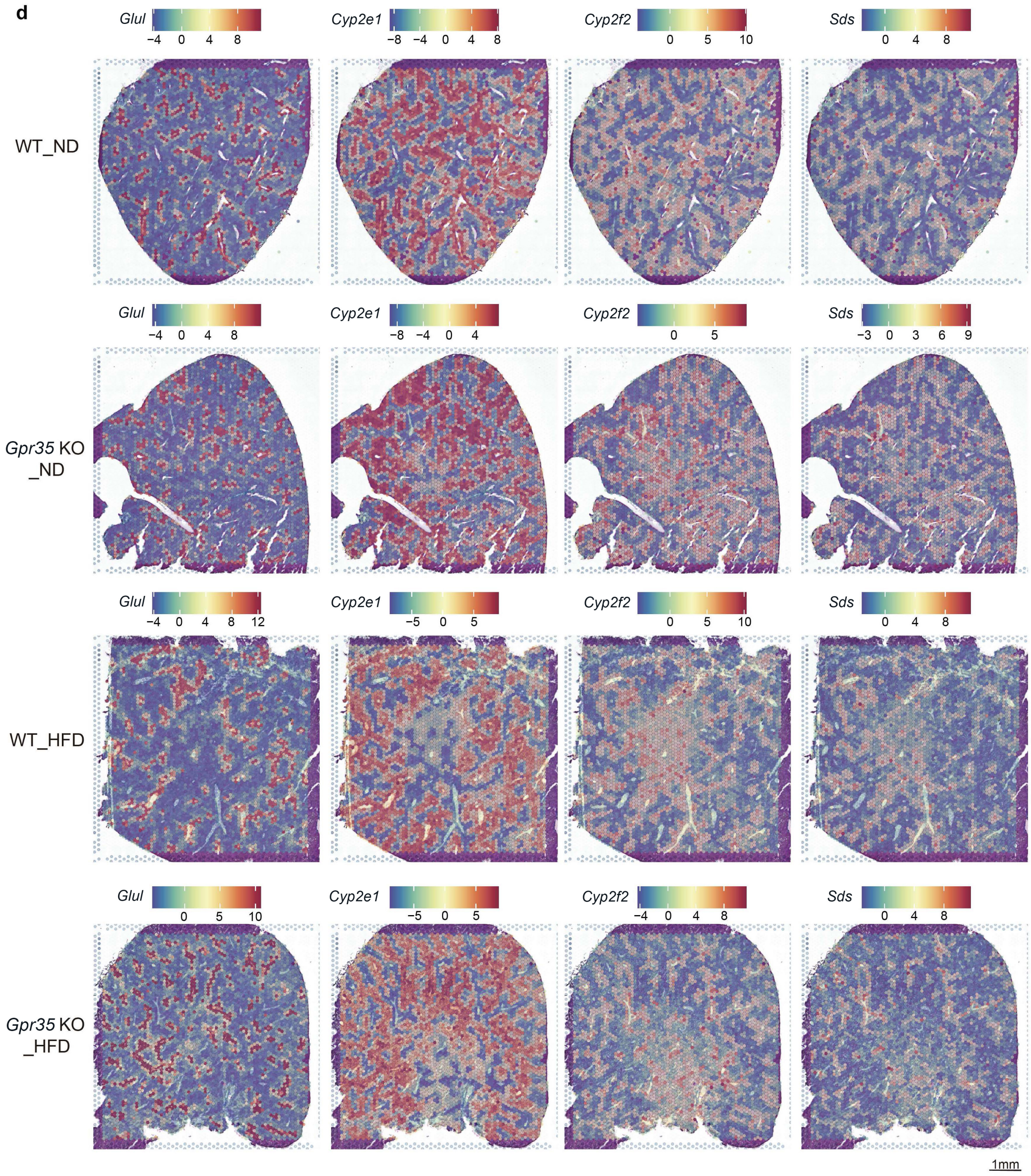

Supplementary Figure S2g

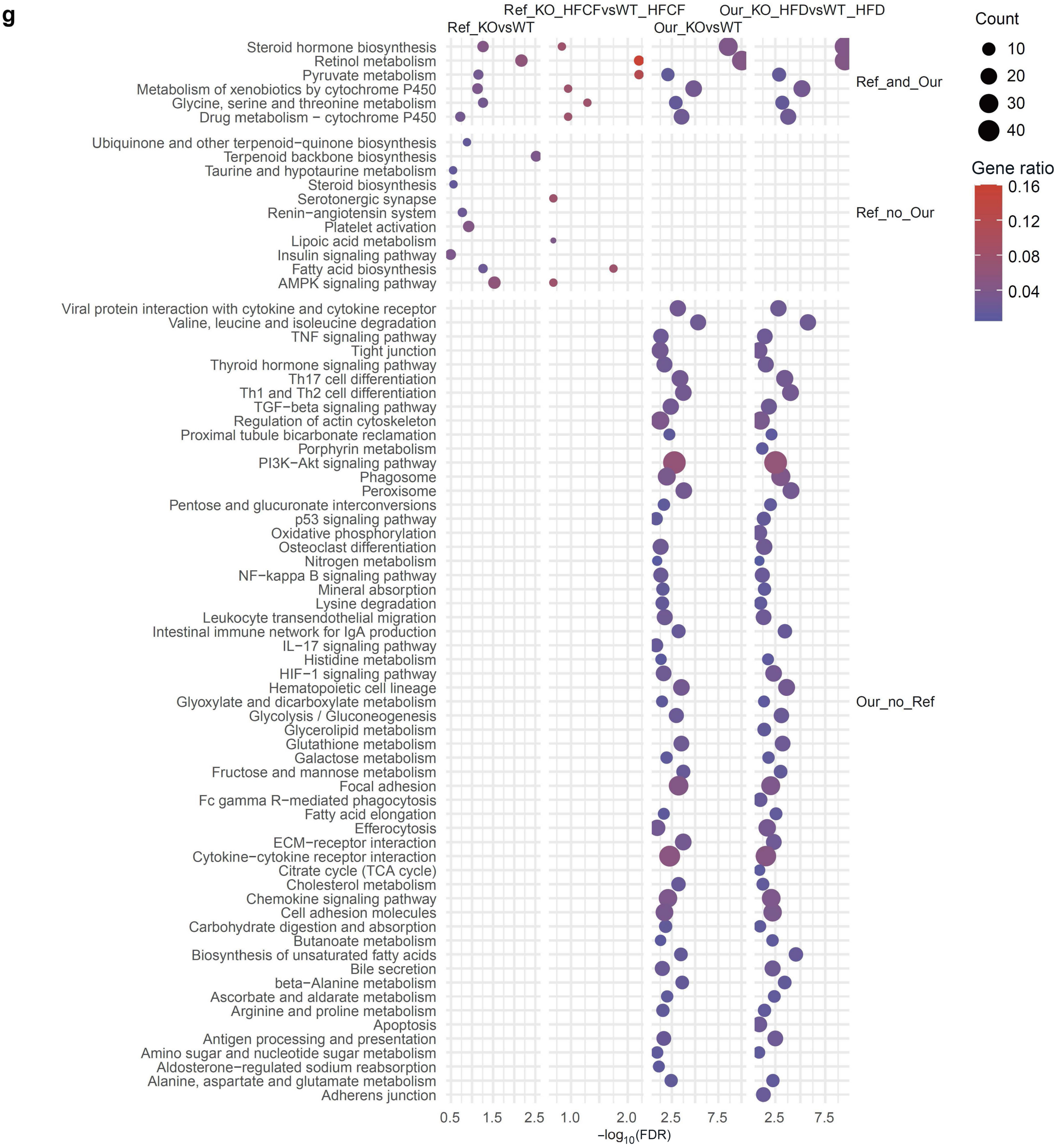

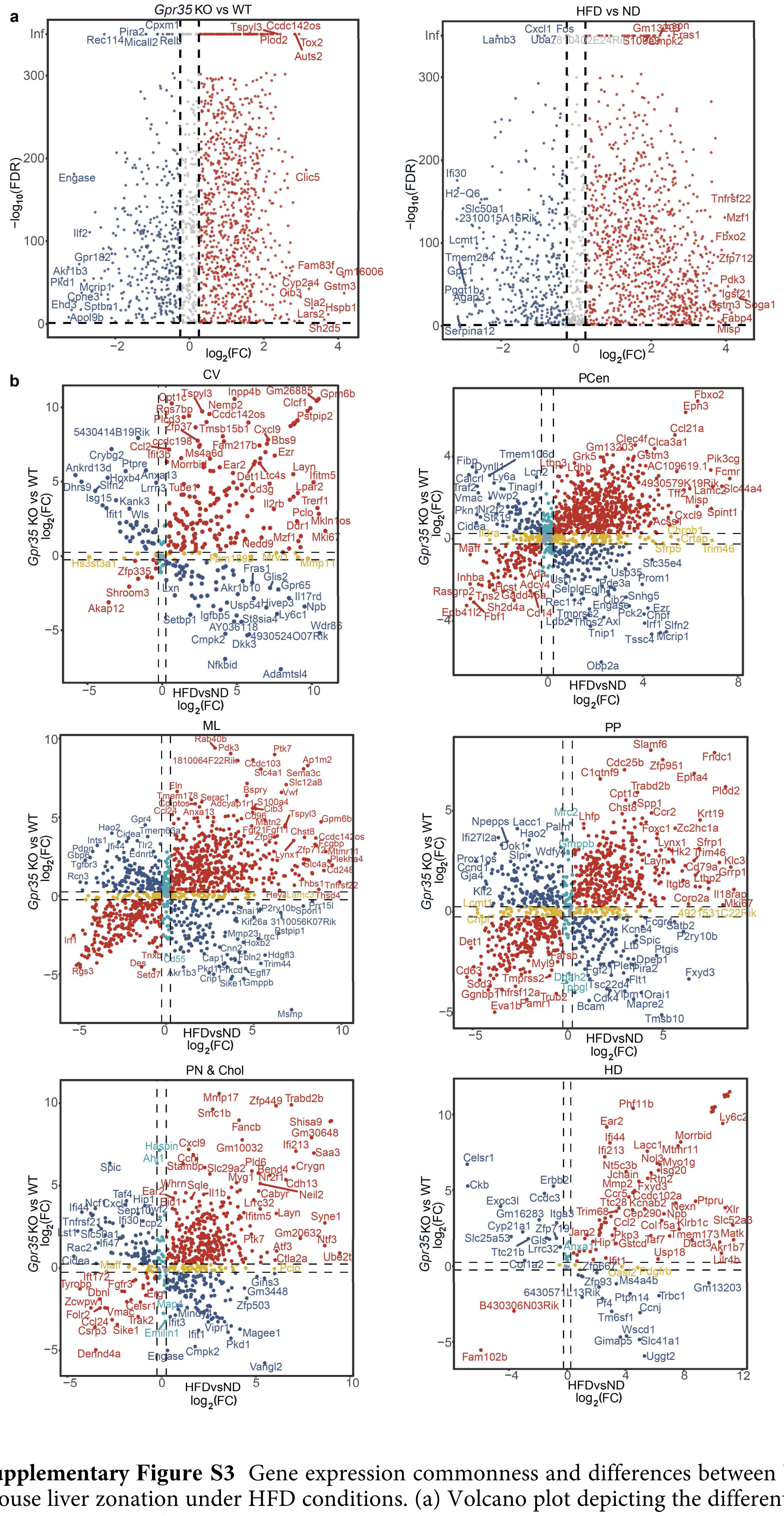

Supplementary Figure S3 Gene expression commonness and differences between WT and *Gpr35* KO mouse liver zonation under HFD conditions. (a) Volcano plot depicting the differential gene expression between samples of *Gpr35* KO and WT, as well as between those subjected to HFD and ND conditions. (b) Scatter plot of DEGs in two different comparison manners with respect to different liver zonation clusters including CV (a), PCen (b), ML (c), PP (d), PN & Chol (e), and HD (f). The X-axis represents the average $\log_2$(FC) (avg_$\log_2$(FC)) computed by comparing the HFD and ND liver tissues (HFDvsND). The Y-axis represents the average $\log_2$(FC) (avg_$\log_2$(FC)) computed by comparing the *Gpr35* KO and WT liver tissues (KOvsWT). The point colors represent the significance types.

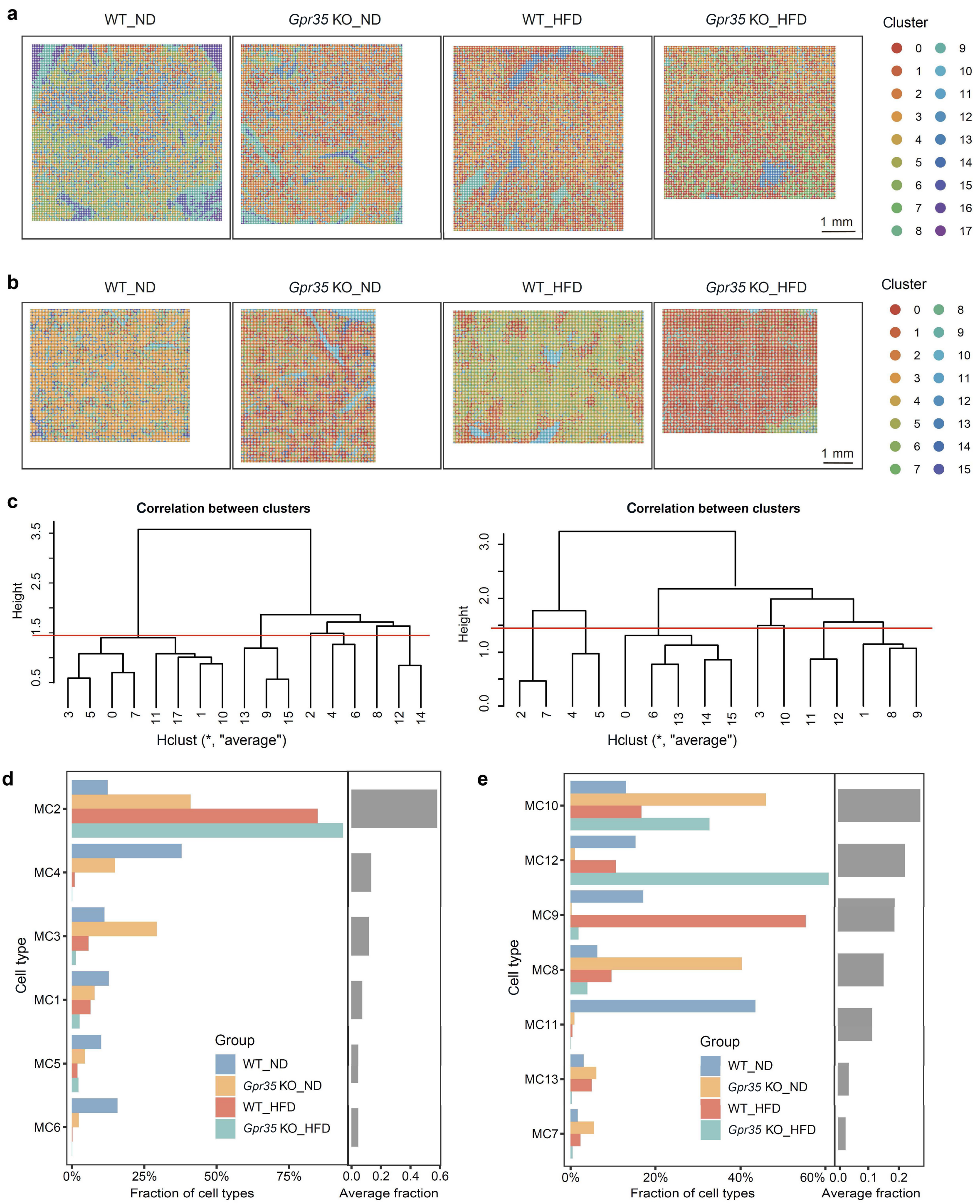

Supplementary Figure S4 Spatial metabolite features in WT and *Gpr35* KO mouse liver zonation under ND and HFD conditions. (a and b) Spatial distribution of the SM spot-based initial Louvain clusters in WT and *Gpr35* KO mouse livers under ND and HFD conditions in both negative (a) and positive (b) ion modes. (c) Dendrograms showing the hierarchical clustering results of the original SM-based clusters in negative (upper) and positive (lower) ion modes. The similarity between the initial Louvain clusters were calculated with the euclidean distance measure of clusters (mean expression of metabolites that were detected in all four samples). The red line marks the selected cut line for separating the final MCs. (d and e) Quantification of the negative ion (d) and positive ion (e) mode MC compositions in different samples. The Y-axis represents MC, and the X-axis represents the percentage. The colors represent sample types.

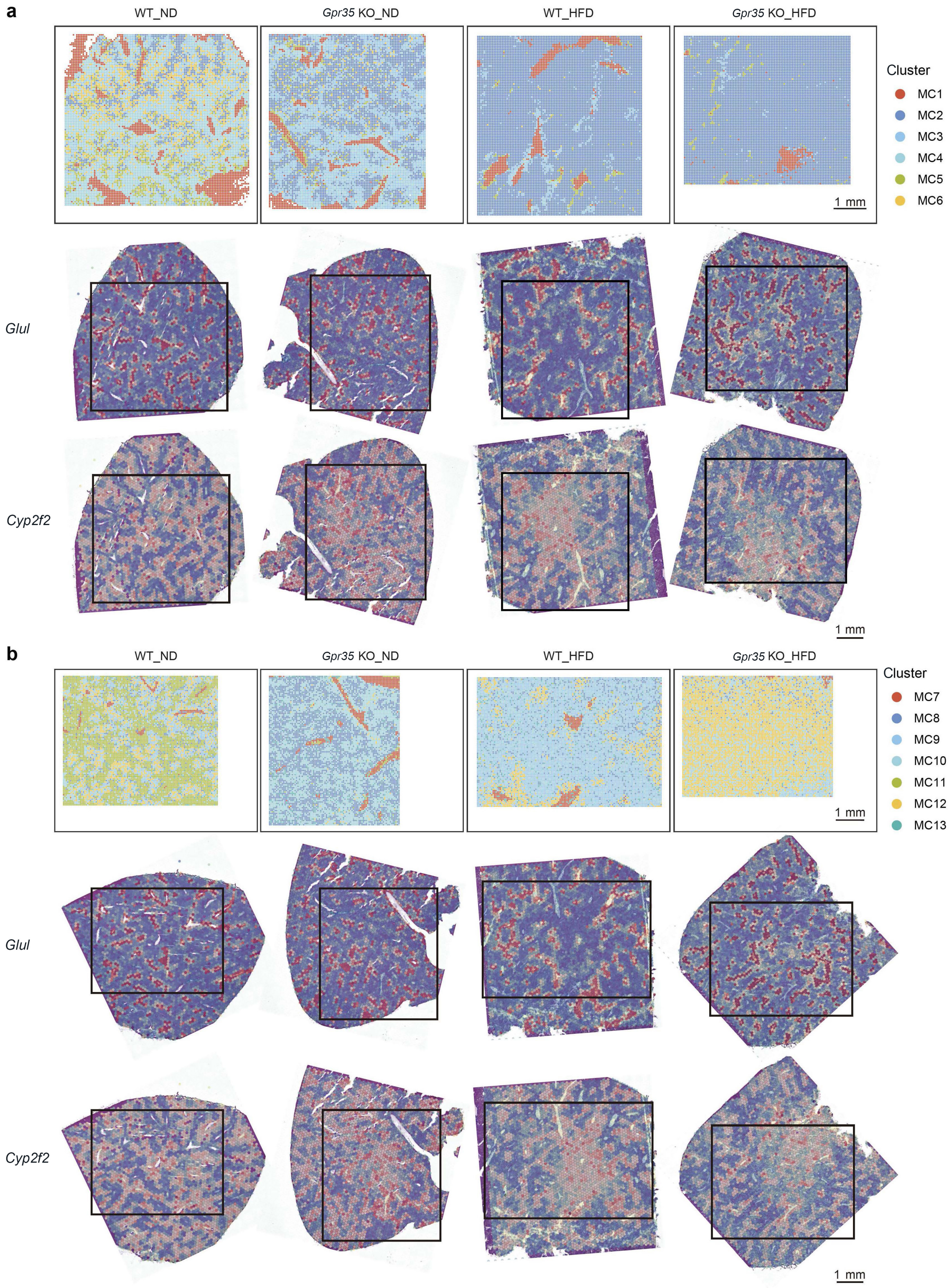

Supplementary Figure S5 Mapping of metabolite clusters with liver zonation marker genes in WT and *Gpr35* KO mouse liver zonation under ND and HFD conditions. (a and b) Spatial distribution of the MCs in WT and *Gpr35* KO mouse livers under ND and HFD conditions in both negative (a) and positive (b) ion modes. Below the SM data, the corresponding regions in the ST profiles are mannully marked and the spatial expressions of *Glul* and *Cypl2f2* are displayed.

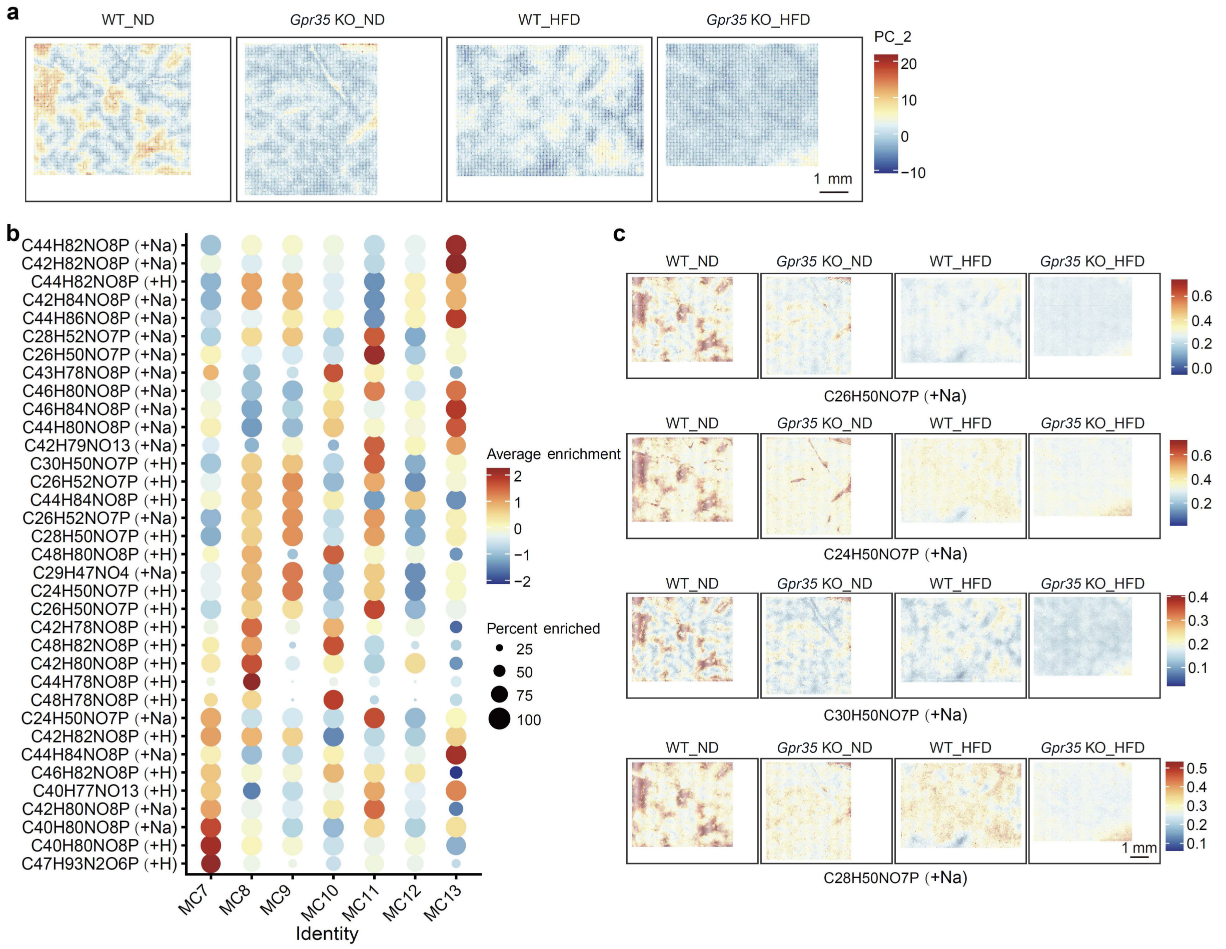

Supplementary Figure S6 Spatial distribution of phospholipids in WT and *Gpr35* KO mouse livers under ND and HFD conditions. (a) Spatial distributions of the first PC determined by the positive ion mode in the four different liver tissue samples. (b) Dot plot of phospholipid enrichment in different MCs. The colors and point sizes reflect average enrichment and enriched percentage of specific phospholipids in each cluster. (c) Spatial distribution of C26H50NO7P, C24H50NO7P, C30H50NO7P, and C28H50NO7P in WT and *Gpr35* KO mouse livers under ND and HFD conditions. These metabolites were specifically distributed in Zone 1 area.

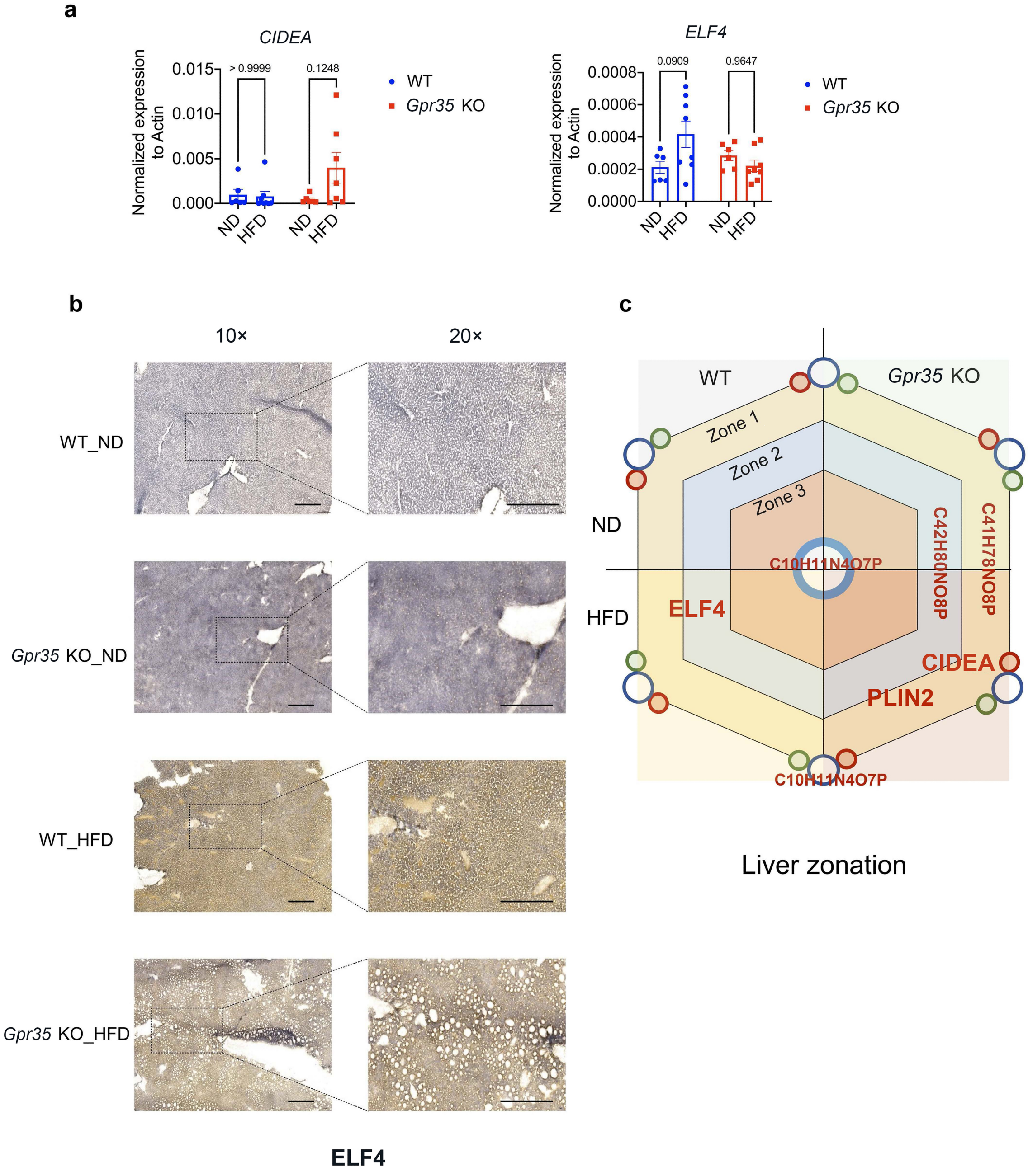

Supplementary Figure S7 CIDEA and ELF4 are potential downstream regulators of GPR35 in MASLD. (a) qRT-PCR analysis of *Elf4* and *Cidea* in WT or *Gpr35* KO mouse liver tissues under ND or HFD conditions (n = 6 for ND, n = 8 for HFD). (b) Representative image of IHC staining of ELF4 in WT or *Gpr35* KO mouse liver under ND or HFD conditions. Scale bar, 100 μm. (c) Diagram of metabolites and genes are discovered in this study.

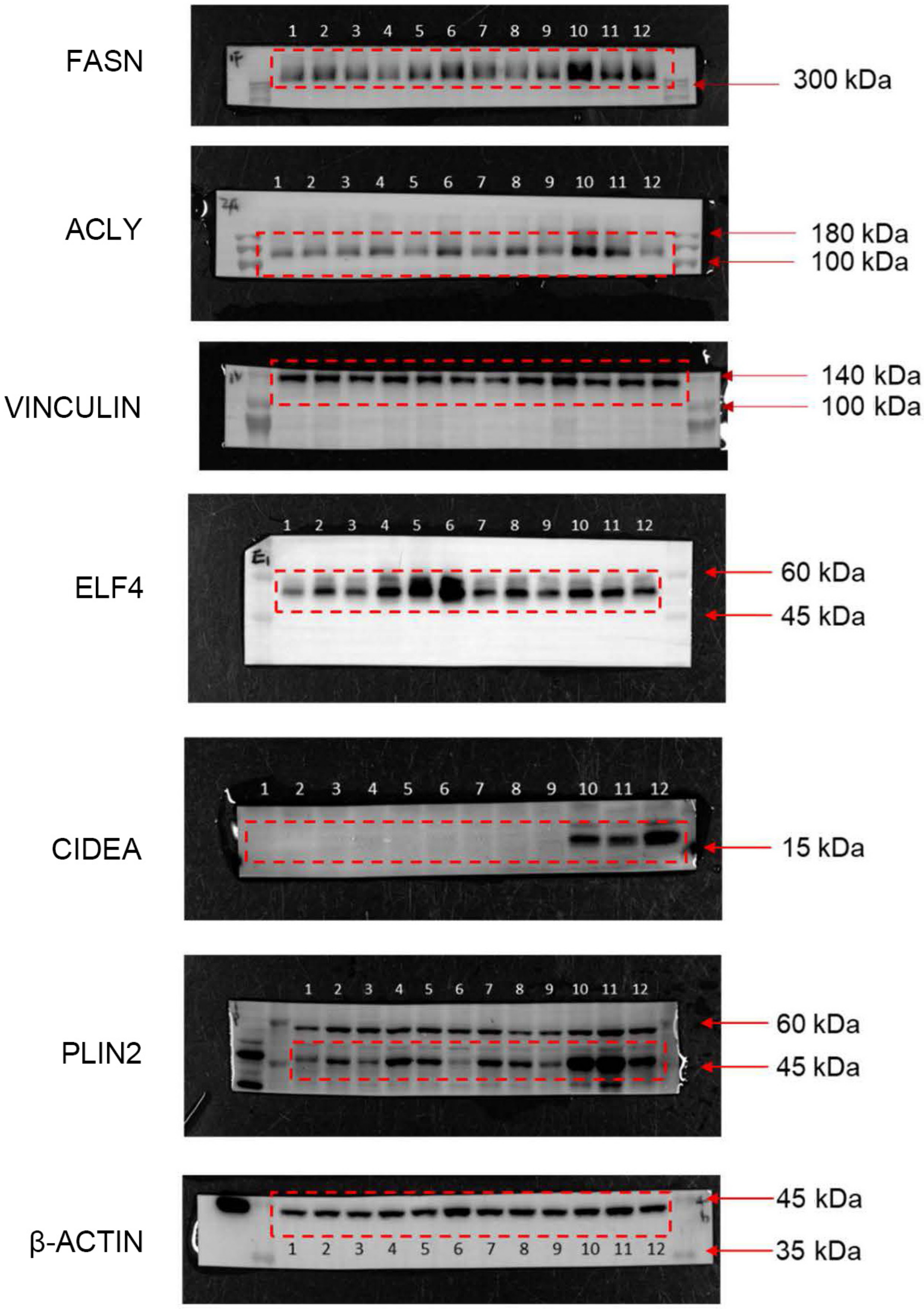

Supplementary Figure S8 Uncropped immunoblotting images.
